# The Role of Branch Cell Symmetry and Other Critical Nanoscale Design Parameters in the Determination of Dendrimer Encapsulation Properties

**DOI:** 10.3390/biom10040642

**Published:** 2020-04-21

**Authors:** Donald A. Tomalia, Linda S. Nixon, David M. Hedstrand

**Affiliations:** 1 Department of Chemistry, University of Pennsylvania, Philadelphia, PA 19104, USA; 2 Department of Physics, Virginia Commonwealth University, Richmond, VA 23284, USA; 3 National Dendrimer and Nanotechnology Center, NanoSynthons LLC, 1200 N. Fancher Avenue, Mt. Pleasant, MI 48858, USA; linda.nixon@nanosynthons.com (L.S.N.); david.hedstrand@nanosynthons.com (D.M.H.)

**Keywords:** dendrimer branch cell symmetry, symmetrical/asymmetrical branch cells, dendrimer drug solubilization, unimolecular micelles, dendrimer symmetry categories, drug encapsulation

## Abstract

This article reviews progress over the past three decades related to the role of dendrimer-based, branch cell symmetry in the development of advanced drug delivery systems, aqueous based compatibilizers/solubilizers/excipients and nano-metal cluster catalysts. Historically, it begins with early unreported work by the Tomalia Group (i.e., The Dow Chemical Co.) revealing that all known dendrimer family types may be divided into two major symmetry categories; namely: Category I: symmetrical branch cell dendrimers (e.g., Tomalia, Vögtle, Newkome-type dendrimers) possessing interior hollowness/porosity and Category II: asymmetrical branch cell dendrimers (e.g., Denkewalter-type) possessing no interior void space. These two branch cell symmetry features were shown to be pivotal in directing internal packing modes; thereby, differentiating key dendrimer properties such as densities, refractive indices and interior porosities. Furthermore, this discovery provided an explanation for unimolecular micelle encapsulation (UME) behavior observed exclusively for Category I, but not for Category II. This account surveys early experiments confirming the inextricable influence of dendrimer branch cell symmetry on interior packing properties, first examples of Category (I) based UME behavior, nuclear magnetic resonance (NMR) protocols for systematic encapsulation characterization, application of these principles to the solubilization of active approved drugs, engineering dendrimer critical nanoscale design parameters (CNDPs) for optimized properties and concluding with high optimism for the anticipated role of dendrimer-based solubilization principles in emerging new life science, drug delivery and nanomedical applications.

## 1. Introduction

It is common knowledge that branch cells are critical architectural components defining the interiors of all dendrimers and dendritic polymers. Furthermore, intrinsic dendrimer branch cell features are determined by specific branch cell reagent structural properties involved in a dendrimer construction. Such features include the following: elemental compositions (i.e., carbon/hetero-atom ratios), carbon hybridizations, hydrophobic/hydrophilic character, aromatic/aliphatic features. To date, over fifty distinctly different dendrimer family types have been reported based on these parameters; wherein, unique and specific properties associated with these family types have defined a broad range of nanomaterial and life science applications. That withstanding, virtually nothing has been reported on the role that dendrimer branch cell symmetry features might play in the determination of dendrimer-based container properties related to the phenomenon of unimolecular micelle encapsulation (UME) behavior. This account will trace the progress and insights developed over the past 35 years concerning the role that dendrimer-based branch cell symmetry has played in the current development of new drug delivery systems, advanced aqueous-based compatibilizers/solubilizers/excipients, dendrimer-based nano-catalysts and metal nano-cluster synthesis.

### 1.1. Architectures Leading to Supramolecular, Guest–Host Encapsulation Properties

**Figure 1:**
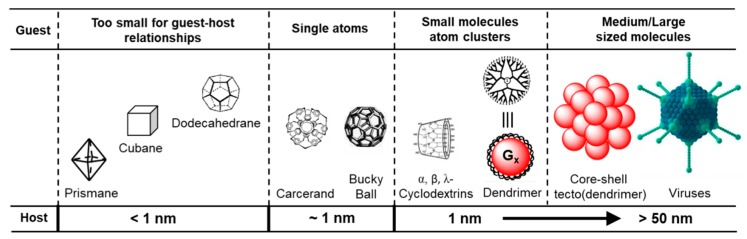
Evolution of closed, container-like structures as a function of dimensions and complexity.

The evolution of closed platonic architectures/organic structures leading to “container-type” molecules manifesting guest–host encapsulation properties may be traced back to the original synthesis of cubane [1], pentaprismane [2], and dodecahedrane [3] (Figure 1). These earlier structures all possessed interiors too small to host either organic or inorganic guest molecules. In 1983, however, Cram et al., [4] reported the first synthesis of carcerand, which was a closed hydrocarbon large enough to host simple organic structures, inorganic ions, or gases. This seminal discovery led to a vast array of similar container-type structures and has been reviewed extensively elsewhere [5]. As early as 1891, container structures derived from oligosaccharides and resembling truncated cones were described by Villiers [6]. These structures, now referred to as α, β or γ–cyclodextrins were not studied extensively until the 1980s; wherein, their encapsulation complexes were extensively reviewed in 1998 [7].

### 1.2. Emergence of Dendrimers and the Dendritic State 

Nearly simultaneous with Cram’s work (1979–1982), the first total synthesis of two high-molecular-weight, mono-dispersed poly(amidoamine) (PAMAM) dendrimer families was demonstrated using the divergent strategy. These early divergent syntheses were based on ammonia and ethylenediamine (EDA) cores, respectively, and completed by the Tomalia Group [8,9,10] in the Dow Chemical Research Laboratories [11]. This effort involved a 12-step iterative synthesis/ characterization of NH_3_ core, PAMAM dendrimers (i.e., generations 0–5), giving discrete molecular weights as high as 10,617 Da. Whereas, a 16-step synthesis of EDA core, PAMAM dendrimers (i.e., generations 0-7), produced discrete molecular weights as high as 57,972 Da. Although official Dow approval for peer reviewed publication was delayed several years (1979–1984), many of these early seminal observations were allowed to be reported publicly before our first peer reviewed publication [12]. This occurred at an invited Pauling-Flory Polymer, Gordon Conference [13], as well as at a variety of academic lectures [14,15,16]. At that time, there was intense Dow management interest/curiosity in how these unprecedented dendritic macromolecular products might fit into their existing commercial polymer business. As such, Dow approval for oral presentations was granted to provide a conduit as possible outside dialogue/feedback concerning potential applications for these dendritic polymers. In fact, it was at the Pauling–Flory Winter Gordon Conference (1983) in Santa Barbara, CA that it was first revealed that Denkewalter et al. (i.e., Allied Chemical Co.) had just been granted a USA Patent [17] for a similar dendritic macromolecular architecture involving an α-aminoacid-based poly(peptide) synthesis. To our surprise, this recently granted Denkewalter patent was considered prior art and essentially all pending Dow patent applications related to dendrimers/dendritic macromolecules were rejected. As stated by the USA patent examiners, none of the Dow patent applications would ever be granted unless it could be demonstrated unequivocally that there were new and unexpected physico-chemical property differences not shared by the two related Denkewalter and Tomalia dendritic polymer architectures. This serious patent dilemma led to several critical comparative studies. These studies ultimately demonstrated the critical role of dendritic branch cell symmetry, as well as its significant influence on dendrimer properties and are overviewed in the next section. That withstanding, many lecture Q&A sessions associated with efforts to obtain external feedback on potential applications focused on the inexplicable PAMAM dendrimer behavior. In many cases, observations suggested some undefined guest–host type encapsulation properties. For example, why do chloroform solutions of ester-terminated PAMAM dendrimers readily dissolve copper salts to produce homogenous, transparent blue solutions; whereas, in the absence of PAMAM dendrimer the salts are completely insoluble? Why is it difficult to remove certain organic compounds containing carboxylic acid, phenolic or hydrogen bonding moieties from PAMAM dendrimers bearing benign/neutral surface groups? These inexplicable observations ultimately led to extensive experimental studies that demonstrated the important role of dendrimer branch cell symmetry in determining guest–host encapsulation properties as described later. 

### 1.3. First Divergent, Iterative Synthesis of High Molecular Weight Dendritic Structures Beyond Vögtle’s Cascade Molecules

As early as 1978, Vögtle et al. [18] described the divergent, iterative synthesis of low-molecular-weight “cascade molecules” which were obtained by Michael addition of amines to acrylonitrile followed by reduction of the nitrile moieties with Rainey-cobalt/H_2_. This original process was unable to produce a dendritic structure higher than 1000 Daltons due to low yields and incomplete reactions at the reduction step. However, 15 years later, independent work by Mulhaupt [19] and Meijer [20] improved the original Vögtle process sufficiently to provide commercial quantities of macromolecular poly(propyleneimine) (PPI) dendrimers (G0–5). This process is now generally referred to as the Vögtle/Mulhaupt/Meijer process. 

#### 1.3.1. Denkewalter-Type Dendrimer Synthesis

**Scheme 1:**
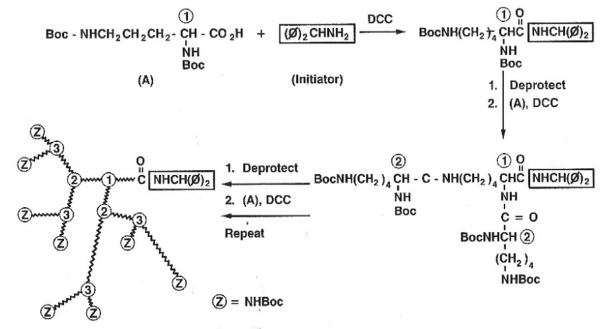
First divergent synthesis of asymmetric branch cell Denkewalter-type poly(lysine) (PL) dendrimers (G0–10) using iterative traditional polypeptide protect–deprotect reaction sequences leading to [Core: diphenylhydrylamine]; *dendri-*poly(amido)-(NH_2_)_Z_;(G0–10) (PL) dendrimers. Reprinted with permission from [10]. Copyright 1990 John Wiley & Sons.

The first macromolecular dendritic structures possessing *asymmetric branch cells* were reported by Denkewalter et al. in a USA Patent, which was granted in 1981 [17]. This patent described the synthesis of an asymmetric branch cell, poly(peptide) dendrimer series based on L-lysine building blocks. Using traditional poly(peptide) synthesis protocols, poly(L-lysine) (PL) dendrimers (G0–10) were obtained with molecular weights as high as 300 kDa, as illustrated in Scheme 1.

#### 1.3.2. Tomalia-Type Dendrimer Synthesis

**Scheme 2:**
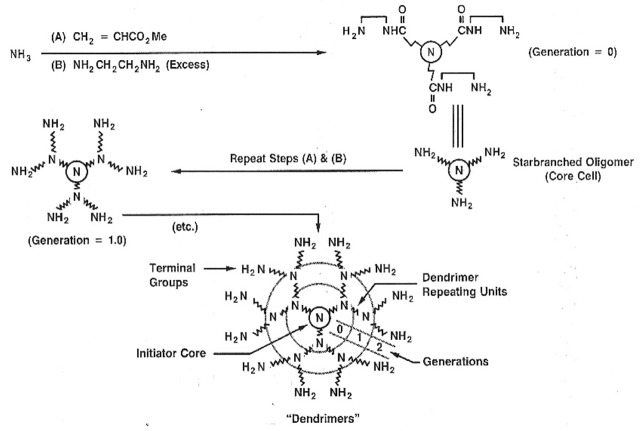
First divergent synthesis of symmetrical branch cell Tomalia-type poly(amidoamine) (PAMAM) dendrimers (G0–7) using iterative reaction sequences (A), (B) leading to [Core:NH_3_]; *dendri*-poly(amidoamine); (-NH_2_)_3-12_; (G1–3); PAMAM dendrimers [11]. Reprinted with permission from [10]. Copyright 1990 John Wiley & Sons.

The first macromolecular dendritic structures possessing symmetrical branch cells were reported orally by Tomalia et al. at the Pauling–Flory Winter Polymer Gordon Conference and described in a publication by Prof. P.-G. de Gennes [21]. Subsequently, this work was presented at the 1st SPSJ International Polymer Conference, Kyoto, Japan, Abstracts (1984); Lecture (1985), where the term “dendrimer” was first introduced by Tomalia. This work described the synthesis of poly (amidoamine) (PAMAM) dendrimers using an iterative two-step process involving: (1) Michael addition of methyl acrylate to an amine followed by (2) amidation with excess ethylene diamine to give a symmetrical branch cell, poly(amidoamine) (PAMAM) dendrimer series (G0–7) with molecular weights as high as 47 kD, as described in Scheme 2. This work was subsequently published in peer-reviewed journals [11,22] and as a USA Patent [23]. 

#### 1.3.3. Newkome-Type Dendrimer Synthesis 

**Scheme 3:**
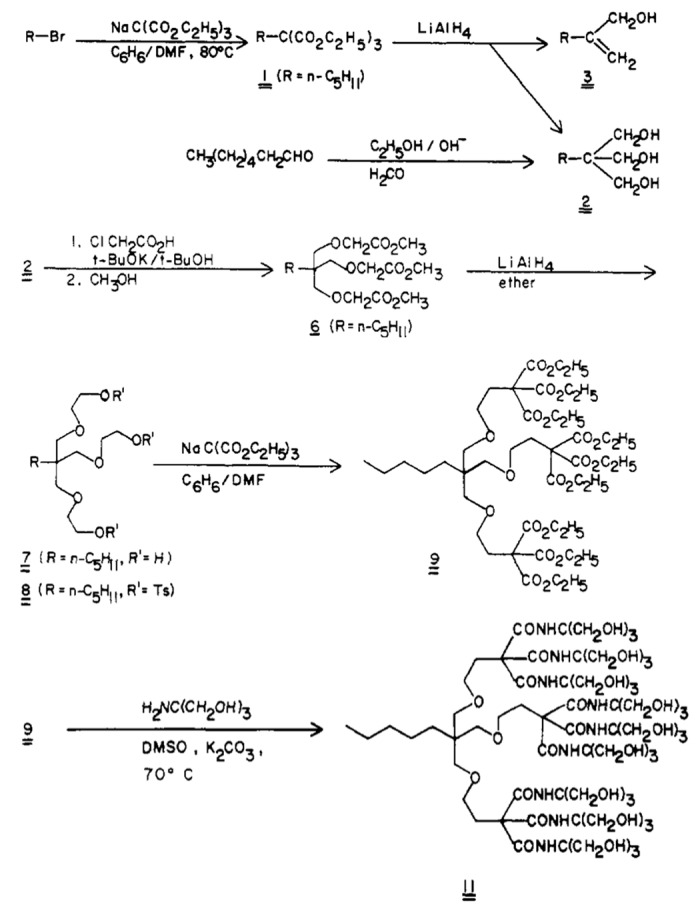
First divergent synthesis of symmetrical branch cell Newkome-type poly(amidoethers) using iterative reaction sequences leading to [core: pentane]; *dendri*-poly(amidoether); (-OH)_Z=3-27_; (G1–3) dendrons referred to as arborols. Printed with permission from [25]. Copyright 1985 American Chemical Society.

The first divergent, dendritic synthesis (i.e., [27]-arborol]) reported by Newkome et al. [24] was actually a series of [core:pentane focal point functionalized]; *dendri-*poly(etheramide);(G1-3);(–OH)_3-27_ dendrons. These divergent, iterative reactions involved sequential (1) ether and (2) amidation steps to produce a G3 dendron, which amplified surface groups as a power function of 3. This produced a dendritic surface moiety enhancement sequence of (i.e., 3→9→27), as shown in Scheme 3, which were referred to as “arborols”. The molecular weight of the final dendritic structure was less than 2000 da (i.e., MWt. ~1910) and not considered to be a dendritic macromolecular structure as reported by Denkewalter (i.e., G0–10) and Tomalia (i.e., G0–7). 

Based on simplified NMR spectra obtained for these dendrons, Newkome proposed these arborol structures possessed symmetrical topology and might represent a new approach to micelles. That withstanding, it was unclear whether Newkome meant the combination of focal point alkyl tail and the dendritic head were reminiscent of classical amphiphilic surfactants or that the dendritic head was a spheroid reminiscent of a covalently fixed, unimolecular micelle. Shortly thereafter, first experimental evidence was reported by Tomalia et al. [25] confirming that PAMAM dendrimers behaved as unimolecular micelles and in fact exhibited unequivocal unimolecular micelle encapsulation (UME) behavior by encapsulating guest molecules such as common aspirin and the agricultural herbicide; 2,4-dichloropenoxy acetic acid with loading levels as high as ~30 wt.% [26].

## 2. Divergent Dendrimer Syntheses Leading to Symmetry Differentiated Branching Topologies

After the first reports by Denkewalter and Tomalia describing the successful synthesis of dendritic macrostructures based on iterative, divergent branching principles, it was assumed that these two generic synthesis strategies did indeed produce equivalent dendritic architectures and branch cell topologies. Without careful introspection, this flawed assumption led to initial rejections of nearly all early Dow Chemical Co. dendrimer patent applications filed during 1980−1984. However, this assumption was soon corrected and led to the first Dow/Tomalia composition of matter dendrimer patent allowance in 1985. It occurred after it was discovered that Tomalia-type PAMAM dendrimers and Denkewalter-type PL dendrimers possessed different symmetry properties and exhibited completely different physico-chemical properties when compared as a function of generation level. In fact, it was the direct comparison of generational properties for these two dendrimer types that revealed the unique “nanoperiodic property patterns” now referred to as dendritic effects [27].

### 2.1. Symmetrical Versus Asymmetrical Branch Cell Dendrimers

**Figure 2:**
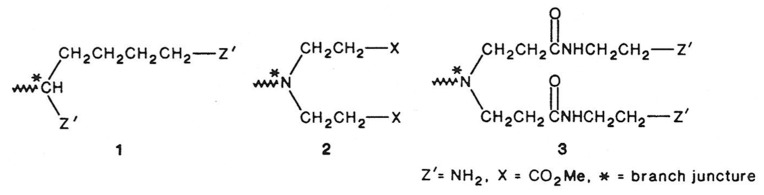
Branch cell (**1**); asymmetrical unequal segmented Denkewalter-type poly(L-lysine) (PL) dendrimers. Branch cells (**2**) and (**3**) symmetrical, equal segmented Tomalia-type poly(amidoamine) (PAMAM) dendrimers. Reprinted with permission from [28]. Copyright 1987 American Chemical Society.

Early opinions assumed that since Denkewalter PL dendrimers and Tomalia PAMAM dendrimers were obtained by similar divergent, iterative branching principles that they would be expected to produce comparable dendritic architectures. However, closer scrutiny of the respective branch cell building blocks (i.e., branch cell monomers) involved in each synthesis clearly revealed critical differences in symmetry and branch cell segment lengths. More specifically, the lysine building block used in the Denkewalter synthesis (i.e., structure (**1**)), Figure 2 is asymmetric and possesses unequal branch segments; whereas, the two Tomalia building blocks (i.e., structures aminoester (**2**) and amidoamine (**3**)), Figure 2, leading to PAMAM dendrimers are symmetrical and possess equal branch cell segments.

**Figure 3:**
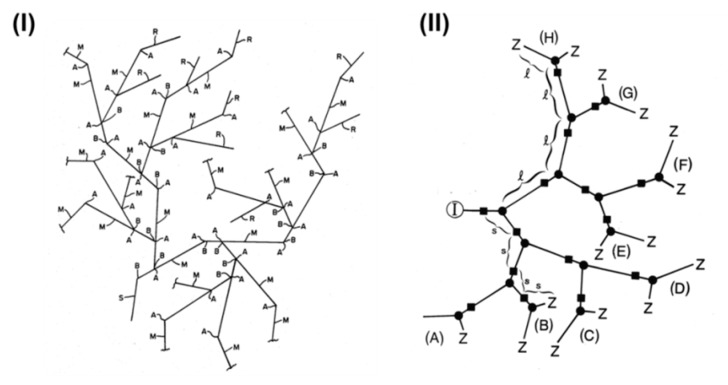
(**I**) Two-dimensional projection of a monodendron with asymmetrical branch junctures (i.e., Denkewalter topology) as it appeared in the Denkewalter patent [17]. (**II**) Two-dimensional projection of a Denkewalter monodendron with surface branch cells (A–H). The (s) and (l) notations are the short and long segment length, respectively. Reprinted with permission from [10]. Copyright 1990 John Wiley & Sons.

**Figure 4:**
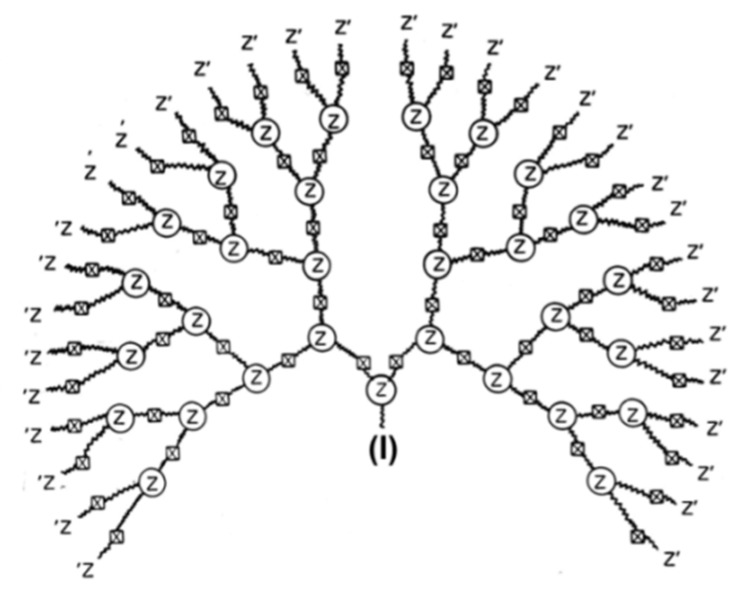
Two-dimensional projection of a monodendron with symmetrical branch junctures (i.e., Tomalia topologies) and Z = branch cell junctures with equal branch segments. Reprinted with permission from [28]. Copyright 1987 American Chemical Society.

As such, Denkewalter PL dendrons (Figure 3) and Tomalia PAMAM dendrons (Figure 4) and their corresponding dendrimers are expected to exhibit significant differences in branch cell topologies. As illustrated in Figure 3, unequal branch cell segment lengths (structure I) associated with the Denkewalter L-lysine building blocks lead to asymmetrical branch cells.

Considering the asymmetrical features of Denkewalter topologies, suggests the involvement of more efficient packing events leading to compact motifs lacking interior void space. On the other hand, it may be hypothesized that the symmetrical, equally segmented branch cells in Tomalia-type dendrimer topologies might lead to less efficient assembly and extended packing motifs that could accommodate the formation of solvent-filled interior void spaces. If true, this subtle change in branch cell symmetry should be expected to impose a dramatic topologic influence on major and measurable physical properties. Major dendrimer properties expected to be affected would include dendrimer densities, refractive indices and hollowness (i.e., ability to encapsulate guest molecules) as a function of generation level. Success in demonstrating such major property differences between Denkewalter and Tomalia architectures would provide critical evidence for overcoming dendrimer composition patent rejections for all pending Dow applications filed 1981–1985 due to the Denkewalter patent allowance in 1981 [17], which was considered prior art.

### 2.2. Significant Property Differences Observed for Denkewalter Poly(lysine) Versus Tomalia Poly(amidoamine) Dendrimers 

**Figure 5:**
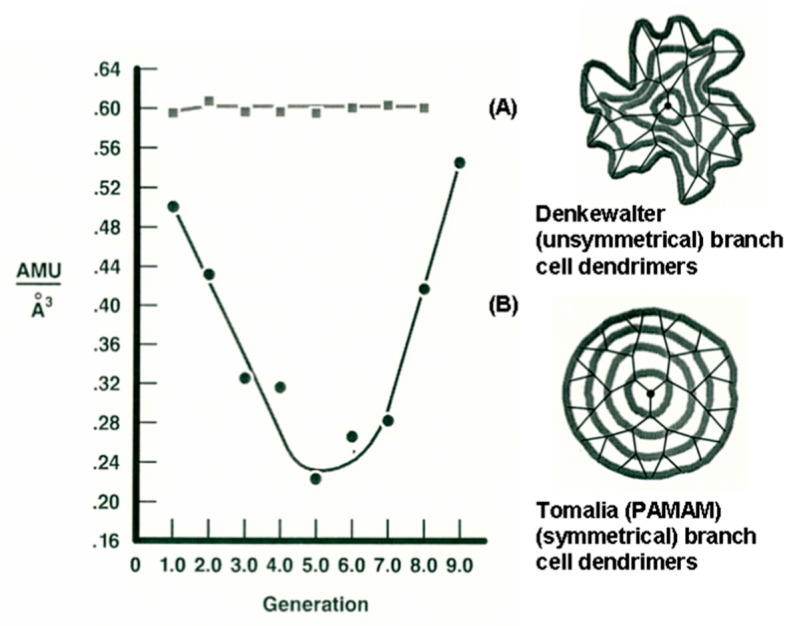
Comparison of densities as a function of generation for (**A**) asymmetrical branch cell in Denkewalter-type dendrimers, (**B**) symmetrical branch cell in Tomalia-type dendrimers (densities calculated from experimental and theoretical hydrodynamic diameters) [28]. Copyright 2012 Reproduced by permission from [27]. Copyright 2012 The Royal Society of Chemistry (RSC) on behalf of the Centre National de la Recherche Scientifique (CNRS) and the RSC.

Inspired by this patent dilemma, a systematic examination of major physical property differences (e.g., densities) as a function of generation was conducted for Denkewalter and Tomalia dendrimers, respectively. Quite remarkably, this study revealed that unequally segmented, asymmetrical branch cell topology associated with Denkewalter PL dendrimers, exhibited a constant density value as a function of generation (i.e., G1–8) in Figure 5A. In contrast, the Tomalia-type PAMAM dendrimers possessing equal segmented, symmetrical branch cell topology exhibited a well-defined, monotonic decrease in density (i.e., G1–4) with a minimum at G4. This downward trend was then followed by a monotonic increase in density from G4–8, as illustrated in Figure 5B.

Recognizing that refractive index values are mathematically dependent on substrate densities, it was not surprising to find that refractive indices for Denkewalter dendrimers remained constant as a function of generation (i.e., G1-8). In contrast, Tomalia-type PAMAM dendrimers exhibited refractive index values that paralleled the variable density values (Figure 5B) with a minima at G4 [10]. These data provided strong evidence for the presence of solvent-filled void space within the interior of Tomalia-type PAMAM dendrimers. On the other hand, the Denkewalter protein-type dendrimers appear to exclude development of such interior void space. This is very likely due to more efficient interior packing associated with the asymmetrical, unequal segmented lysine branch cells. These results were further confirmed by Aharoni et al. [29] who showed that Denkewalter PL dendritic macromolecules were indeed dense, non-draining spheroids exhibiting no evidence of hollowness or shell-like topology as a function of generation/molecular weight.

**Figure 6:**
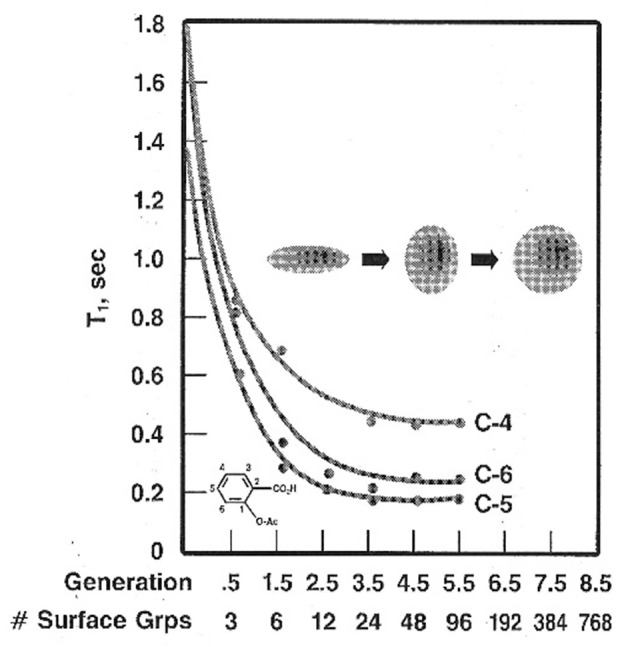
A comparison of spin lattice relaxation times for acetylsalicylic acid carbons (i.e., C-4, -5 and -6) combined with [core:EDA]; *dendri*-(poly(amidoamine)(-CO_2_Me)_Z=3-96_; (G0.5-5.5) PAMAM dendrimers as a function of generation and # of surface groups.

Further corroboration of this hypothesis occurred in 1989; wherein, supramolecular encapsulation of small organic guest molecules such as aspirin and 2,4-dichlorophenoxy acetic acid was demonstrated [25,26,28]. This work was based on observed reductions in ^13^carbon spin lattice relaxation times as a function of PAMAM generation level for certain guest molecules (i.e., 2, 4-dichlorophenoxyacetic acid), as described in Figure 6.

**Figure 7:**
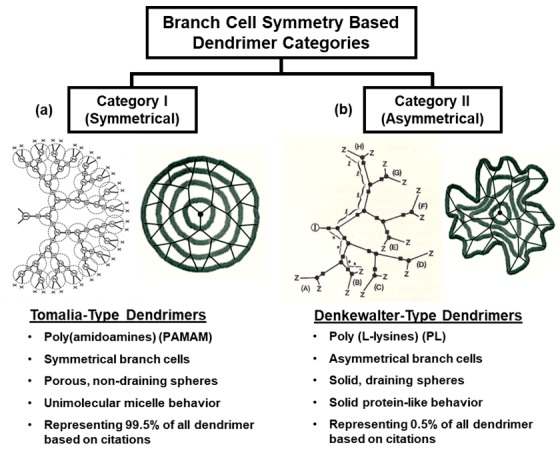
Classification of first divergently synthesized dendrimers (**a**) Denkewalter poly(L-lysine) (PL) dense draining spheroids; (**b**) Tomalia, poly(amidoamine) (PAMAM) hollow, non-draining spheroids (1984).

Ultimately, the strong evidence described above, forced all international patent offices and examiners to formally redefine and classify the first two divergently synthesized dendrimers into two distinctly different topology categories; namely: (I) symmetrical branch cell and (II) asymmetrical branch cell topologies, as shown in Figure 7. In essence, the Denkewalter and Tomalia dendrimer topologies were recognized and defined as two distinctly different and novel patentable compositions, each of which exhibit intrinsically different physical properties due to their uniquely differentiated topologies. Based on this unified patent perspective, allowance was soon granted for essentially all Tomalia/Dow symmetrical branch cell type patents; wherein, the list includes >51 USA patents, as well as all equivalent worldwide patents. It is noteworthy, that there are currently no known examples of host–guest encapsulation involving Denkewalter-type PL dendrimers containing asymmetrical branch cell topology [30]. 

At this time, this highly specialized category of asymmetrical Denkewalter-type dendrimers exhibiting *dense draining spheroidal* properties appears to be confined to only polypeptide/ protein-type dendrimers. These dendrimers are normally produced using traditional protein synthesis protocols; namely, protect–deprotect and solid phase synthesis methodologies. It is interesting to note, that this protein-type dendrimer category (i.e., Denkewalter type) has received substantially less attention in the literature compared to traditional Tomalia and Newkome type, symmetrical branch cell dendrimers. According to a recent review article by Haridas et al. [31], a SciFinder search from 1993 to 2019 reveals only 282 research publications related to asymmetrical branch cell Denkewalter-type dendrimers have appeared in the literature. In contrast, during this same time (i.e., 1990–2019), >50,000 publications have been documented for symmetrical branch cell containing Tomalia-type dendrimers. According to this article, only a handful of protein-type dendrimer examples exist. These protein dendrimers are largely confined to the extensive work by Starpharma Ltd., Melbourne, Australia focused on the use of Denkewalter’s original PL dendrimers for a wide range of life science applications including antivirals, microbicides and targeted cancer therapies (www.starpharma.com).

This equal-segmented, symmetrical branch cell Tomalia-type dendrimer category constitutes the majority of all dendrimer families known to date. It defines and represents the largest know classification of divergently synthesized dendrimers. As such, it is incorrect to describe the Denkewalter dendrimers (i.e., reported incorrectly as patent granted 1981 versus actual patent granted in 1983 [17,32] as the first synthetic example representing all major divergently synthesized dendrimer types. Historically, Denkewalter dendrimers are indeed seminal first examples of highly specialized, asymmetrical, branch cell dendrimer topologies; however, they do not exhibit typical interior properties/behavior manifested by symmetrical-branch cell dendrimer topologies that constitute a preponderance of all dendrimer families known to date. These protein-like dendrimers behave as dense, draining spheroids. As such, Denkewalter dendrimers lack porosity/ hollowness, behaving much like a solid nanoparticle. They do not manifest encapsulation properties associated with all currently known dendrimer families derived from symmetrical branch cell monomers. As a consequence, Denkewalter dendrimer applications are largely associated with only two critical nanoscale design parameters (CNDPs); namely, discrete nanoscale sizes and polyvalent surface chemistries.

Unexpected dendrimer-based guest–host encapsulation properties were first reported in 1989 by Tomalia et al. [26] involving PAMAM dendrimer hosts and small organic guest molecules (i.e., aspirin and 2,4-dichlorophenoxy acetic acid). Similarly, the encapsulation of small inorganic guest structures such as metal salts was reported in 1985 [11,23,33] and for zero-valent metal clusters (i.e., copper, iron, nickel, gold, silver) was first reported in Tomalia patents [23,34] and subsequently, in the literature as early as 1998 [35,36,37,38,39,40,41,42].

### 2.3. Historical Overview: First Divergently Synthesized Dendrimer Topologies Reported by Tomalia, Newkome and Denkewalter

Historically, the first divergent synthesis of low-molecular-weight dendrimer precursors (i.e., <1 KD) was reported by Vögtle et al. [18] and were referred to as cascade molecules. Between 1983 and 1985, three independent groups reported the divergent synthesis of true dendritic macromolecular structures now referred to as dendrimers (i.e., a term first coined by Tomalia et al.) [11,23]. It is notable that two of these three groups performed these dendrimer syntheses in industrial laboratories; namely, Tomalia at the Dow Chemical Co. and Denkewalter at the Allied Chemical Co. In each of these cases, complete dendrimer families consisting of advanced generational levels were synthesized. For example, a generational series of PL dendrimers ranging from G0–10 (i.e., representing molecular weights as high as 300 kDa were reported by Denkewalter et al. [17]; whereas, a generational series of PAMAM dendrimers ranging from G0–7 (i.e., representing molecular weights as high as 47 kDa) were reported by Tomalia et al. [11,23]. Meanwhile, an academic group led by Newkome, et al. [24] reported the divergent synthesis of a limited series of dendritic poly(amidoether) structures referred to as arborols. This generational series (i.e., G0–3) of arborols represented molecular weights as high as 1.9 kDa.

As noted in Table 1, Tomalia and Newkome-type dendrimers containing symmetrical, equal-branch cell segmented interior topologies have dominated a majority of the activity in the dendrimer field since inception, based on total literature/patent citations. An overview of PAMAM dendrimer covalent and supramolecular syntheses has been recently reported by Peng et al. [43].

Earlier interest in poly(peptide) type dendrimers was documented largely by Tam et al. [44,45] lysine core dendrimers with tetrapeptides and octapeptides were more soluble in water, more stable to proteolysis and less toxic to human cells than their linear polymeric analogs; comparable antimicrobial potency was demonstrated by Jezek et al. [46]. However, recent work by Haridas et al. [31] exhaustively reviewed the area of peptide/protein dendrimers and has clearly revealed significant new interest in this overlooked area of dendrimer science. More specifically, Urbanczyk-Lipkowska [47] have described the synthesis and evaluation of amphiphilic peptide dendrimer as specific antimicrobials effective against drug-resistant bacteria [48]; whereas, Reymond et al. [49,50] reports the structure–activity relationships for a library of single component, well-defined peptide dendrimers, which are equivalent to Lipofectamine L2000 as a transfection vector for siRNA.

That withstanding, substantial commercial interest in poly(peptide)-type (i.e., PL dendrimers has also been noted based on recent nano-medical clinical successes reported by Starpharma, Ltd., Victoria, Austrualia, (www.starpharma.com); wherein, certain PL dendrimers are being marketed and used as antivirals, microbicides (i.e., VivaGel^®^) and targeted cancer therapies (i.e., DEP^®^ products).

## 3. Unimolecular Micelle Guest–Host Encapsulation Associated with Symmetrical Branch Cell Dendrimers

**Figure 8:**
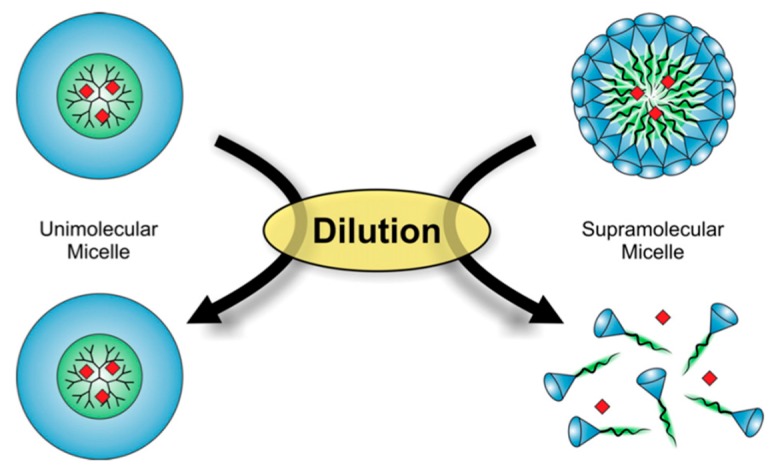
Covalently assembled unimolecular micelles such as symmetrical branch cell dendrimers and traditional multimolecular micelles derived from amphiphilic surfactants. Reprinted with permission from [51]. Copyright 2015 Elsevier.

Traditional micelles involve the use of small-molecule amphiphilic surfactants (i.e., soaps) consisting of hydrophilic heads and hydrophobic tails and are associated with familiar laundry or aqueous cleaning processes. These amphiphilic surfactants form multi-molecular micelles possessing hydrophobic interiors and hydrophilic surfaces, as illustrated in Figure 8. They function by encapsulating and solubilizing hydrophobic (i.e., fatty type) guest molecules within their hydrophobic interiors. These regular micelles are in dynamic equilibrium with their constituent surfactant molecules and form only when a critical surfactant concentration (i.e., critical micelle concentration (CMC)) is present in the aqueous system. Therefore, upon dilution below the CMC, these regular micellar assemblies disassociate back to unassembled surfactant molecules, releasing any captured/encapsulated guest molecules.

**Figure 9:**
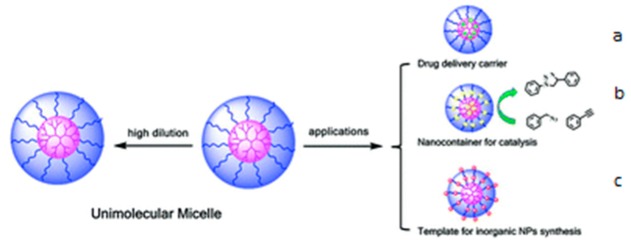
Proposed applications for covalent, unimolecular micelles (**a**) drug delivery, (**b**) catalysis, (**c**) templates for inorganic nanoparticle synthesis. Reproduced with permission from [52]. Copyright 2016 The Royal Society of Chemistry (RSC).

In contrast, unimolecular micelles (Figure 8) are stable, covalently assembled structures that mimic the encapsulation properties of traditional multi-molecular micelles, but do not exhibit dynamic disassociation properties upon dilution. As such, they are of high interest in a range of applications including: (a) drug solubilization/delivery, (b) catalyst and as (c) templates for synthesizing inorganic nanoparticles and metal nanoclusters (Figure 9).

### 3.1. Early Examples of Unimolecular Encapsulation

**Figure 10:**
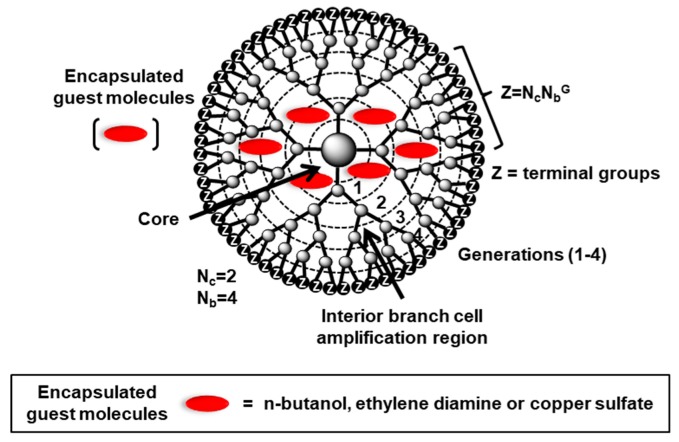
Unimolecular encapsulation of guest molecules (red ellipsoids) in a PAMAM dendrimer host. Where, first observed guest molecule examples = n-butanol, copper sulfate [10]; aspirin, 2,4-dichloro-phenoxy acetic acid [20].

First evidence indicating that PAMAM dendrimers were exhibiting container-like, unimolecular micellar properties was noted in the original dendrimer paper by Tomalia et al. [11]. At that time, it was reported that n-butanol used as an azeotroping agent for the removal of ethylenediamine from the PAMAM dendrimers reactions was readily separated from all PAMAM dendrimer generations, G0-6. However, upon reaching PAMAM G7 it was impossible to remove this azeotroping agent by any means. It was speculated that, n-butanol was entrapped within the interior of the PAMAM dendrimer at that generational level and the surface congestion precluded its removal (Figure 10).

**Figure 11:**
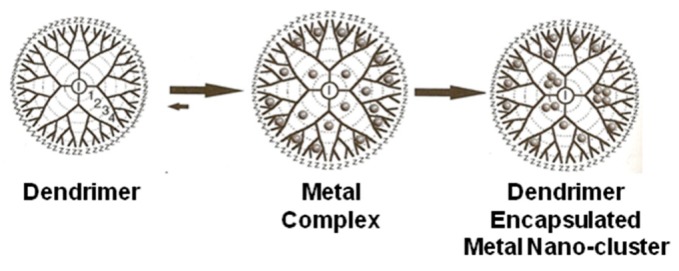
Use of PAMAM dendrimers as ligand functionalized templates for the complexation of metal salts and subsequent reduction to zero-valent metal nanoclusters. Reprinted with permission from [35]. Copyright 1998 American Chemical Society.

Similarly, it was noted that copper salts formed very brilliant blue or purple colored complexes with all ester and amine terminated PAMAM dendrimers, respectively. It was initially speculated that these inorganic salts were penetrating into the PAMAM dendrimer interiors to form these interesting nitrogen-ligand-assisted chromophoric complexes. This was subsequently confirmed by the addition of reducing agents (i.e., ascorbic acid, borohydrides) to these colored metal salt–dendrimer complexes. In 1998, it was reported simultaneously by Tomalia and Crooks that discrete, encapsulated, zero-valency copper metal nanoclusters were formed (Figure 11) [35,36]. Before these simultaneous publications, this unimolecular micelle metal encapsulation behavior had been observed earlier for a wide range of metals as described in the patent literature by Tomalia et al. [23,34,53] and reported extensively elsewhere [10,36,37,38,39,40,41,42].

**Figure 12:**
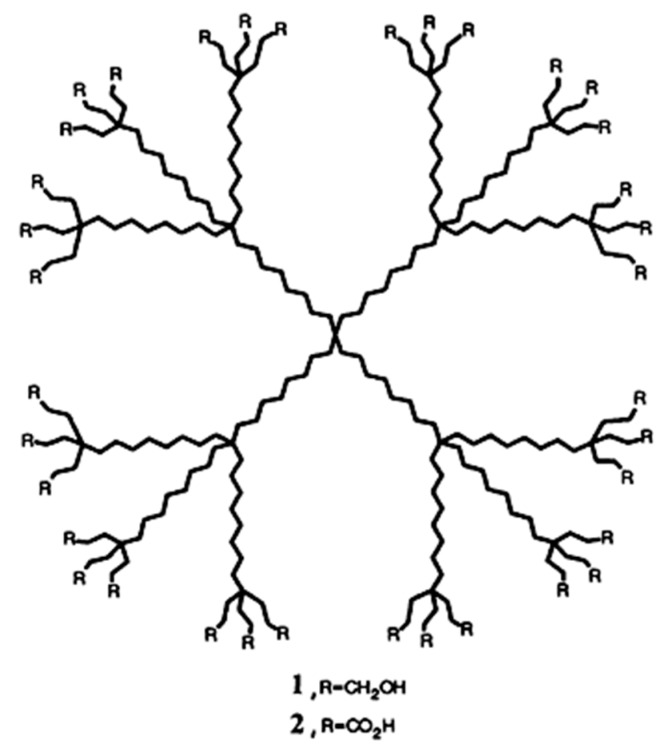
A generation 2, cascade polymer referred to as [8^2^ · 3] micellanoic acid/alcohol exhibited unimolecular micelle behavior. Reprinted with permission from [54]. Copyright 1991 John Wiley & Sons.

Although dendrimers were first proposed to be reminiscent of regular traditional micelles by Newkome [24] and Tomalia [25], this hypothesis was not demonstrated experimentally until 1989 [26]. However, subsequent work in 1991 by Newkome et al. [54,55] provided additional support for the unimolecular micelle concept. It involved the synthesis of a cascade polymer (G2), referred to as [8^2^ · 3] micellanoic acid, possessing hydrophobic interior and hydrophilic surface moieties as illustrated in Figure 12. This unimolecular prototype provided additional evidence by encapsulating a series of small hydrophobic guest molecules (i.e., diphenylhexayriene, phenol blue, naphthalene).

**Figure 13:**
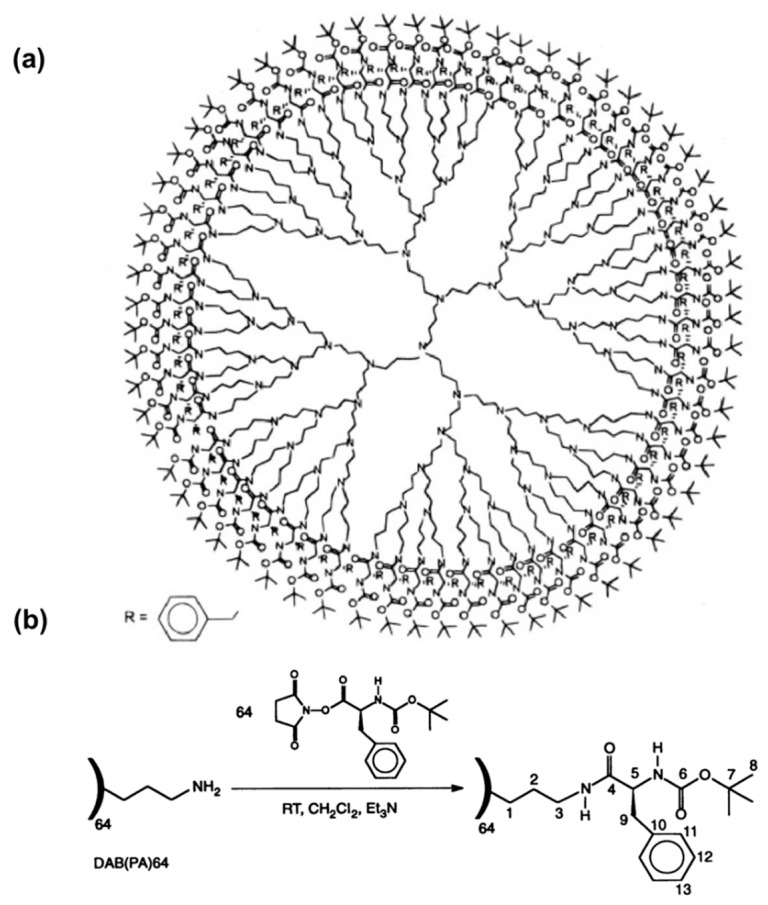
(**a**) Structure of a DAB core, (G4), boc-protected, α-aminoacid-terminated PPI dendrimer referred to as a dendritic box and (**b**) synthesis of the shell defining the dendritic box. Reprinted with permission from [57]. Copyright 1995 American Chemical Society.

Essentially, all reported dendrimer-based unimolecular encapsulation examples involved open dendritic structures with surface chemistry that allowed dynamic guest entry and departure from the host. However, Meijer et al. [56] described the unique synthesis of a poly(propyleneimine) (PPI) dendrimer modified with α-aminoacid surface chemistry (Figure 13a) that was demonstrated to sterically entrap guest molecules such as Rose Bengal or 7,7,8,8 tetracyano-quinodimethane (TCNQ) and not allow departure from their interior. Synthetic modification involved conjugation of a boc-protected α-aminoacid to surface amine groups of PPI dendrimer (G4); -(NH_2_)_64_ (Figure 13a) in the presence of the guest molecules. These surface modified dendrimers referred to as a dendritic box, were ~5 nm diameter nano-containers containing physically incarcerated guest molecules which could not exit the interior unless the surface groups were perturbed by removal [57].

**Figure 14:**
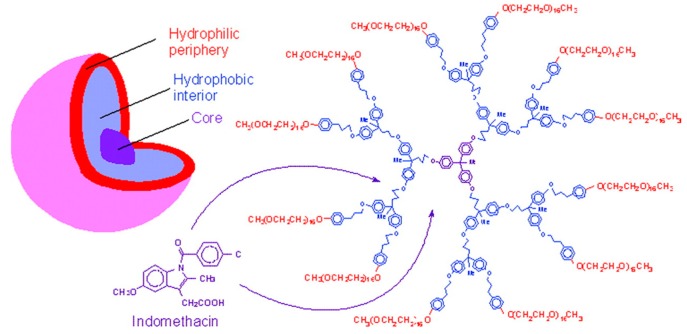
Dendritic unimolecular micelle used for solubilization and controlled release of indomethacin. Reprinted with permission from [58]. Copyright 2000 Elsevier.

Subsequently, Fréchet et al. [58] reported the synthesis of non-nitrogen-containing surface PEGylated dendritic structures derived from large, symmetrical branch celled, hydrophobic hypercores as shown in Figure 14. These unimolecular micelles were shown to solubilize the hydrophobic anti-inflammatory drug, indomethacin, with encapsulation capacities up to 11 wt. % in aqueous solutions.

### 3.2. Brief Overview of Symmetrical, Branch Cell Dendrimer Families Exhibiting Encapsulation Properties

The seminal demonstrations of dendrimer-based unimolecular encapsulation behavior during the 1990s, led to extraordinary activity during the following decades (2000–2020) by applying these principles to many practical applications. Foremost, was the extensive use of a broad range of dendrimer types as water solubilizing agents for a number of water insoluble, active pharmaceutical ingredients (APIs), many of which were FDA-approved drugs.

#### 3.2.1. Early Examples of Unimolecular Micelle Encapsulation

**Figure 15:**
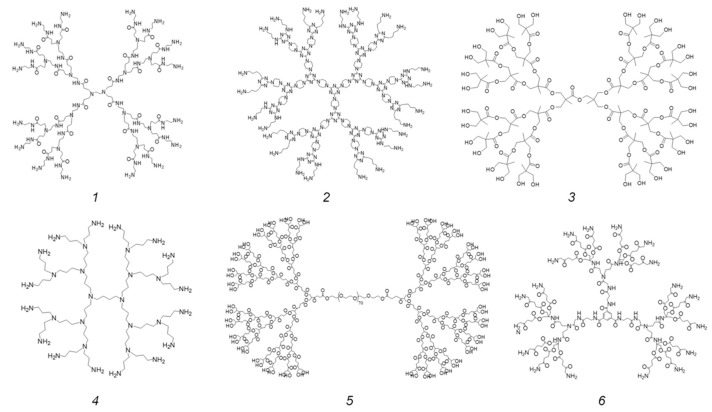
Structures of symmetrical branch cell dendrimer families that have been used for encapsulation of organic guest molecules or active pharmaceuticals (AP); (**1**) poly(amidoamine) (PAMAM), (**2**) triazine-based dendrimers, (**3**) poly(ester) dendrimers based on 2,2′-bis (hydroxylmethyl) propionic acid, (**4**) poly (propylenimine) (PPI), (**5**) dendrimer based on glycerol and succinic acid with a PEG core, (**6**) dendrimer based on 5-amino levulunic acid. Reprinted with permission from [59]. Copyright 2008 Elsevier.

Over the last two decades, there has been enormous interest in the solubilization of both organic and inorganic guest molecules using dendrimer-based unimolecular encapsulation principles. A broad range of symmetrical branch cell dendrimer families were used with typical dendrimer types, as shown in Figure 15.

**Figure 16:**
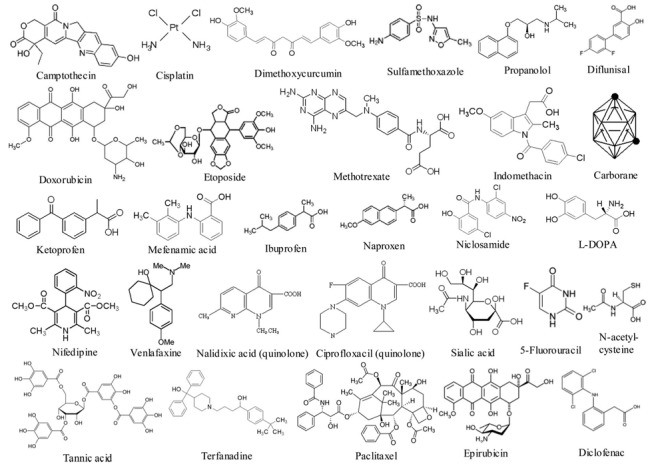
Molecular structures of drugs either encapsulated or stabilized by dendrimers as of 2010. Reprinted with permission from [60]. Copyright 2010 American Chemical Society.

A partial list of guest molecules that were encapsulated, solubilized or stabilized by dendrimers was reviewed by Astruc et al. [60] in 2010, as shown in Figure 16.

**Figure 17:**
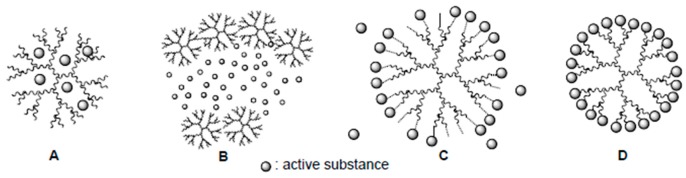
(**A**) Non-covalent unimolecular micelle dendrimer encapsulation, (**B**) non-covalent dendrimer aggregate encapsulation, (**C**) non-covalent dendrimer surface complexation and (**D**) covalent conjugation to the dendrimer surface. Reprinted with permission from [61]. Copyright 2001 Elsevier.

These aqueous guest (drug) solubilization examples were hypothesized to occur by one or more host interaction modes as described in Figure 17A, non-covalent unimolecular micellar dendrimer encapsulation; Figure 17B, non-covalent encapsulation via dendrimer aggregation; Figure 17C, non-covalent dendrimer surface complexation; Figure 17D, covalent conjugation to dendrimer surface. These solubilization events may occur independently by any one of these four modes or in combination.

This activity was documented extensively in many of the following references [58,62,63,64,65,66,67,68,69,70,71]. However, in some cases, guest solubilization involved specific covalent conjugation of a pharmaceutically active agent to the dendrimer surface. Although mentioned later in Section 3.4.1, it will not be a focus of this article.

#### 3.2.2. Factors Affecting Dendrimer Modified Drug Solubilization

**Figure 18:**
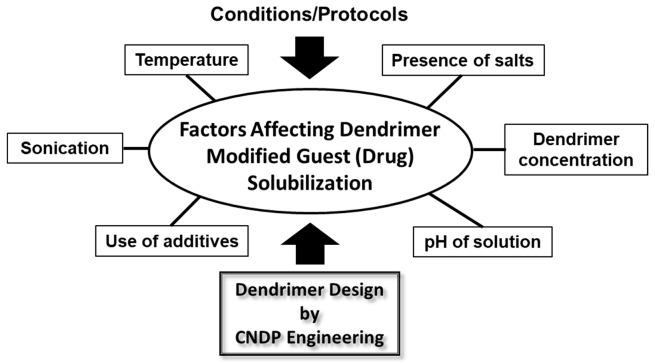
Factors affecting dendrimer modified guest (drug) solubilization conditions/protocols and dendrimer structural design by CNDP engineering.

This early work revealed a number of important factors that determined and affected dendrimer-modified drug solubilization processes. These factors included the importance of: (a) general protocols/conditions used in the solubilization and (b) specific designed/optimized structures obtained by engineering dendrimer critical nanoscale design parameters (CNDPs), as described in Figure 18. Although dendrimer design by CNDP engineering will be discussed more specifically later in Section 5, it should be mentioned that dendrimer interior compositions determine and profoundly affect the ability of a dendrimer to exhibit unimolecular micelle encapsulation of inorganic salts and metals. Generally speaking, dendrimer interior compositions suitable for inorganic ligation contain electron-rich nitrogen, sulfur or phosphorous heteroatoms. As such, dendrimer families most suitable for unimolecular encapsulation of metal salts and metal nano-cluster formation include PAMAM, PPI, Yamamoto phenylazomethane-type, Simanek triazine-type or Majoral phosphorous-type containing dendrimers as described in Section 3.2.4.

#### 3.2.3. Selected Examples of Organic Guest (Drug) Encapsulations

**Figure 19:**
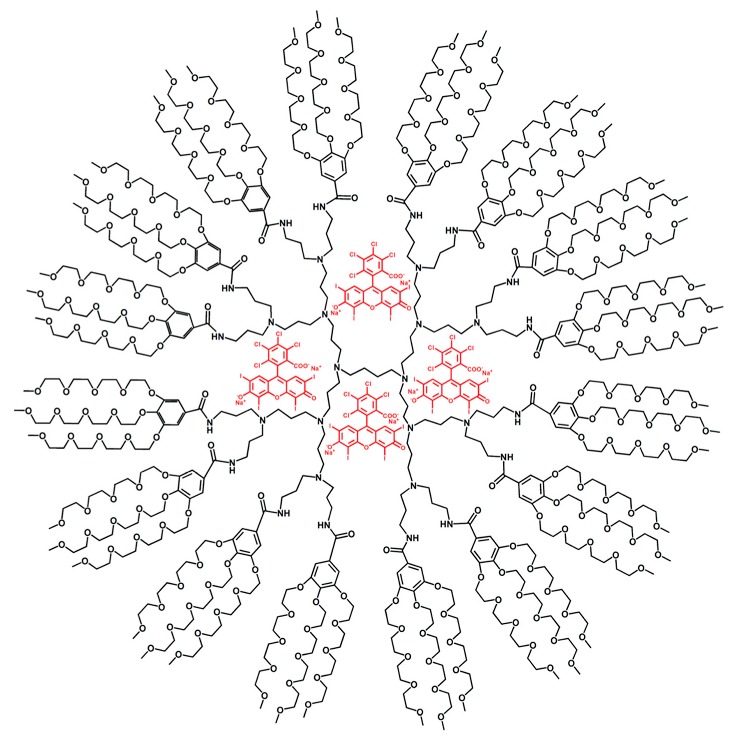
Poly(propylenimine) (PPI) core: PEGylated shell unimolecular micelle containing three molecules of encapsulated Rose Bengal dye. Reprinted with permission from [72]. Copyright 2000 Springer/Nature.

Among some of the earlier examples of dendrimer-based unimolecular micelle encapsulation were reported by Meijer et al. [72]. More specifically, a PPI dendrimer core was modified by conjugating poly(ethylene glycol arms) via the amine surface groups to produce an expanded PEGylated shell structure. These unimolecular micelles were shown to readily encapsulate Rose Bengal as illustrated in Figure 19.

Unarguably, one of the most important early examples describing unimolecular micellar encapsulation of active drugs was reported by Grinstaff et al. [73]. This work involved the unimolecular encapsulation and controlled release of three anti-cancer camptothecins, using a symmetrical branch cell, non-nitrogen containing, poly(ester) dendrimer derived from glycerol and succinic acid (PGLSA). Cellular uptake and efflux measurements in MCF-7 cancer cells demonstrated a 16-fold enhancement in cellular uptake and increased drug retention within the cancer cells when using this dendrimer vehicle. It is notable, that this unimolecular micelle encapsulation vector does not contain any interior nitrogen ligands (i.e., tertiary amines).

**Figure 20:**
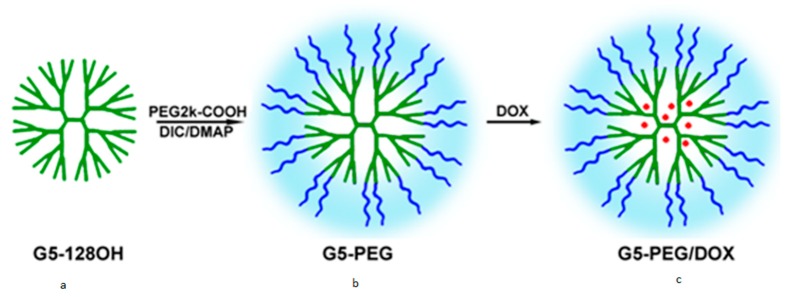
(**a**) Hydrophobic (G5) *dendri-*poly(ester)-(OH)_128_ core, (**b**) [Core: *dendri-* poly(ester)- (OH)_128_;(G5)]-Shell [PEGylated] structure, (**c**) [Core: *dendri-*poly(ester)-(OH)_128_;(G5)]-Shell [PEGylated] structure with encapsulated doxorubicin (DOX). Reprinted with permissions from [74]. Copyright 2013 American Chemical Society.

Synthesis of a hydrophobic poly(ester) dendrimer core-hydrophilic PEGylated shell structure (Figure 20b) was described by Ma et al. [74] and found to exhibit unimolecular micelle behavior with a variety of drugs. Firstly, the synthesis of a G5; poly(ester) (-OH)_128_ dendrimer core (G5-128OH) (Figure 20a) involved combining two AB_2_ monomers (i.e., 2,2-bis-(acryloyloxymethyl) propionic acid (ACPA) and 1-thioglycerol) in a sequential manner using thiol/acrylate Michael addition click reactions and esterification. The resulting hydrophobic core was then PEGylated via the hydroxyl surface groups to produce a dendritic-linear architectural copolymer (G5-PEG) (Figure 20b). This biocompatible dendritic-linear copolymer exhibited excellent capacity for encapsulating and controlled release of hydrophobic anti-cancer drugs such as doxorubicin (DOX) to give G5-PEG/DOX (Figure 20c).

It is generally recognized and assumed that PEGylation of drugs or drug carrier/vector will lead to positive drug-delivery properties. That withstanding, it is also very important to assess and understand the accumulative effect/impact on the pharmacokinetics, metabolism and distribution of the drug when specifically combining a PEGylation component with any other entity including a dendrimer component as described above [75].

**Figure 21:**
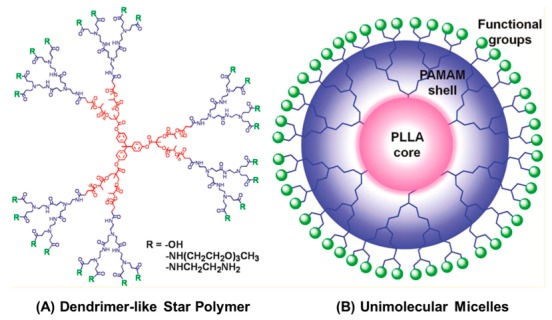
(**A**) Structure of the hydrophobic dendrimer-like star core polymer derived from poly(L-lactide) (PLLA); (**B**) a unimolecular core–shell micelle structure derived from a hydrophobic poly(L-lactide) (PLLA) core and a shell composed of poly(amidoamine) (PAMAM) dendrons. Reprinted with permission from [76]. Copyright 2011 American Chemical Society.

Similarly, Cao and Zhu [76] reported the synthesis of a hydrophobic, star-like poly(L-lactide) core (Figure 21A) followed by the surface conjugation of hydrophilic PAMAM dendrons to produce a hydrophobic core–hydrophilic shell-type amphiphilic structure, as described in Figure 21B. These structures exhibited extraordinary unimolecular micelle encapsulation properties by loading up to 11.5 wt.% of DOX which was readily soluble in aqueous media.

**Figure 22:**
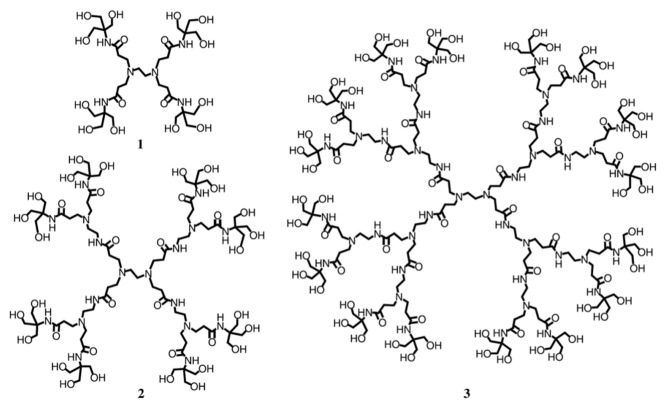
Two dimensional structures (i.e., 1,2,3) of [Core: EDA]; dendri-poly(amidoamine)- (tris-hydroxyl)_4-16_; (G0,1,2); (PAMAM) dendrimers. Reprinted with permission from [62]. Copyright 2003 Elsevier.

Related to earlier work by Tomalia et al. involving aspirin and 2,4-dichloropehenoxy acetic acid [26,77,78], Twyman et al. [62] described the unimolecular encapsulation of hydrophobic organic acids (i.e., benzoic acid) and phenolics (i.e., 2,6-dibromo-4-nitrophenol) into tris-hydroxyl terminated PAMAM dendrimers. It was found, that higher generation (i.e., G1, G2 versus G0), tris-terminated; PAMAM dendrimers were required (Figure 22). It was speculated that interior tertiary amine moieties were involved in acid–base type interactions to facilitate encapsulation. Using calorimetric methods, these authors determined that encapsulated active microbicides or fungicides could be effectively released in controlled therapeutic amounts to inhibit pathogen growth without exhibiting intrinsic toxicity. The importance of the dendrimer generation level (i.e., size, congestion) on catalytic processes involving unimolecular encapsulation events was demonstrated by Twyman et al. [79] and reviewed extensively [80].

**Figure 23:**
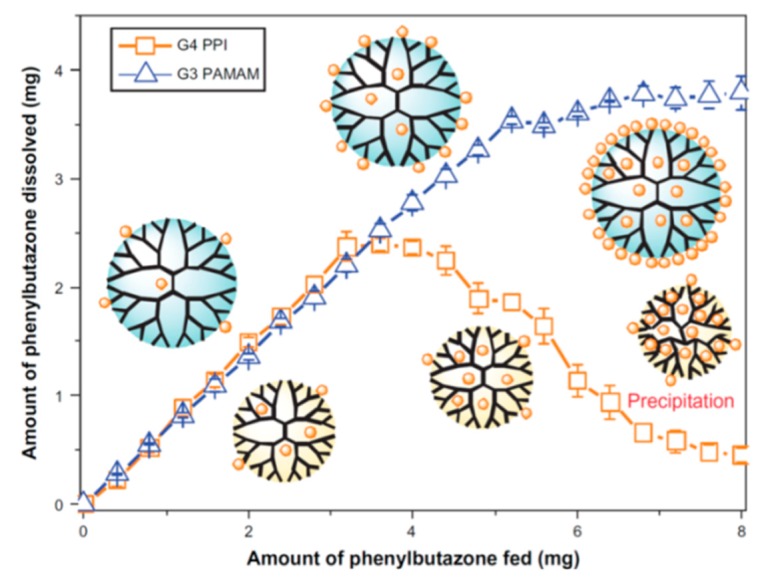
Comparison of drug-loading ability of G3, poly(amidoamine) (PAMAM) and G3, poly(propyleneimine) (PPI) dendrimers using a gradient feed method. Reprinted with permission [81]. Copyright 2011 Dove Medical Press. Note: The authors used old nomenclature and mislabeled the generational levels for PPI dendrimers. See D.A. Tomalia, M. Rookmaker for correct PPI generational nomenclature [82].

It was shown by Cheng et al. [81], that the size of the symmetrical branch cells as well as the nature of nitrogen moieties in the closely related PAMAM and PPI dendrimers, respectively, can dramatically influence drug-loading capabilities of the dendrimeric unimolecular micelle structures involved. A direct comparison of the loading capability of G3 PAMAM and G3 PPI dendrimers with the model drug, phenylbutazone is shown in Figure 23.

**Figure 24:**
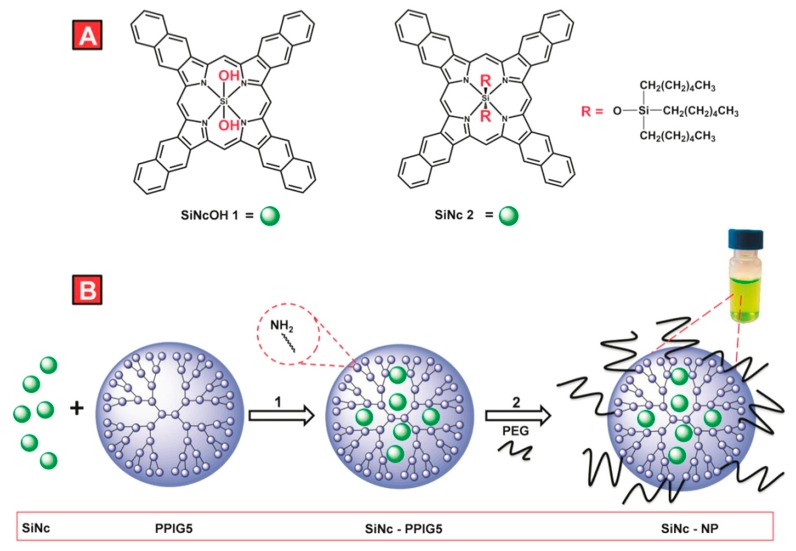
(**A**) Chemical structures of parent silicon naphthalocyanine, silicon 2,3 naphthalocyanine dihydroxide (SiNcOH 1) and substituted derivative, silicon 2,3 naphthalocyanine bis(trihexylsilyloxide) (SiNc 2). (**B**) Schematic illustration of the fabrication of SiNc-loaded theranostic nanoplatform: 1) encapsulation of SiNc molecules within the hydrophobic interior of amine terminated G5 PPI dendrimers (PPIG5); 2) dendrimer surface modification with polyethylene glycol (PEG). The inset represents a photo of SiNc-NP solution in water. Reproduced with permission from [83]. Copyright 2015 The Royal Society of Chemistry.

Interesting work reported by Taratula et al. [83], describes the encapsulation of naphthalocyanine dyes in G5, PPI dendrimers followed by PEGylation of the dendrimer surface to give a single agent, duo-functional unimolecular micellular agent. This simple theranostic device was suitable for concurrent near-infrared (NIR) imaging and therapeutic photodynamic (PDT) treatment of cancer (Figure 24).

#### 3.2.4. Optimal Dendrimer Families for Inorganic Metal Guest Molecules

**Figure 25:**
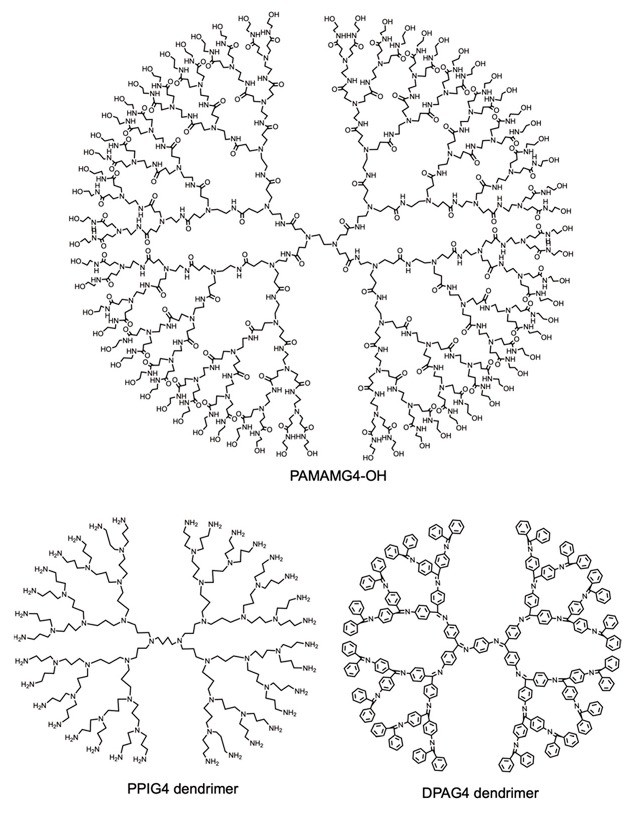
Typical, nitrogen ligand containing, symmetrical branch cell dendrimers suitable for metal salt and metal cluster encapsulations including: (**a**) G4-poly(amidoamine) (PAMAM), (**b**) G3-poly (propylenimine) (PPI) and (**c**) G-4-poly(phenylazomethine) (DPA) dendrimers. Reprinted with permissions from [84]. Copyright 2019 American Chemical Society.

Typical dendrimer hosts shown to be effective for encapsulating metal salt/metal cluster guests usually contain both (a) symmetrical branch cells and (b) electron-rich ligating atoms, as described in Figure 25. It is generally recognized that symmetrical, branch cell dendrimers containing interior nitrogen, phosphorous or other electron-rich hetero-atomic moieties exhibit excellent encapsulation properties for inorganic metal salts. These encapsulation events first involve interior metal salt complex formation. As such, these interior complexation sites may serve as templates for subsequent reduction (i.e., ascorbic acid or borohydride reagents) to produce encapsulated zero-valent metal nano-clusters, as described earlier in Figure 11. Several well-known dendrimer families used for this application are illustrated in Figure 25.

**Figure 26:**
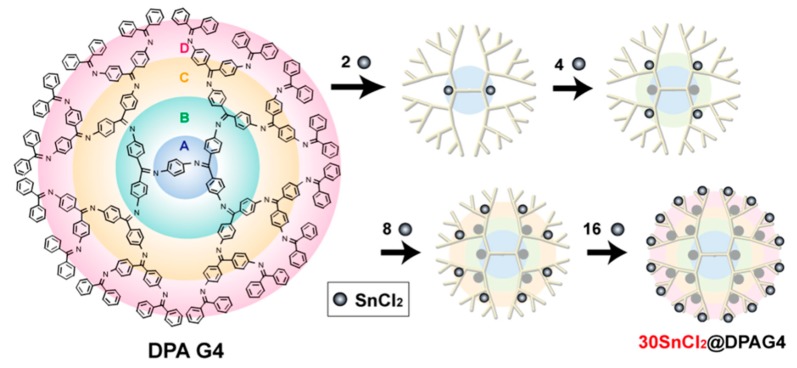
Schematic representation of layer-by-layer stepwise accumulation of metal chloride in phenylazomethine dendrimer (DPAG4). Reprinted with permission from [85]. Copyright 2014 American Chemical Society.

Although this very large and active area of unimolecular metal encapsulation is not the main focus of this article, it is noteworthy that Yamamoto et al. [85] have developed a unique dendrimer family referred to as phenylazomethane dendrimers. Generational member of this dendrimer family may be used as template hosts for the precise interior placement of guest metal atoms, atom by atom, to form discrete metal nano-clusters. Quite surprisingly, these metal encapsulation events are observed to occur stoichiometrically, beginning at the dendrimer core and proceeding outwardly in a well-defined sequence as a function of dendrimer generation. These generation specific ligation events are mathematically predictable as a function of the imine moieties present in each generation and produce well-defined stoichiometries, which are depicted in Figure 26. Subsequent metal reduction within these dendrimer templates produced a variety of precise metal atom nano-clusters, which could be controlled atom by atom. This area has been reviewed by Yamamoto et al., elsewhere [84]; whereas, the general area of dendrimer-based metal encapsulation remains very active and has been reviewed extensively by others [60,86].

It is noteworthy that both the dendrimer core and the branch cell symmetry of certain dendrimers such as an EDA core poly(propyleneimine) (PPI) dendrimer may strongly influence and define specific metal coordination patterns within the dendrimer interior as reported by de la Mata et al. [87]. Furthermore, it was subsequently shown by this group that these specific metal coordination sites could dramatically affect the anti-viral properties exhibited by these metal-modified dendrimers as a function of their scaffolding backbone, generation and peripheral functionality [88].

### 3.3. Influence of Dendrimer CNDPs on Encapsulation Properties

**Figure 27:**
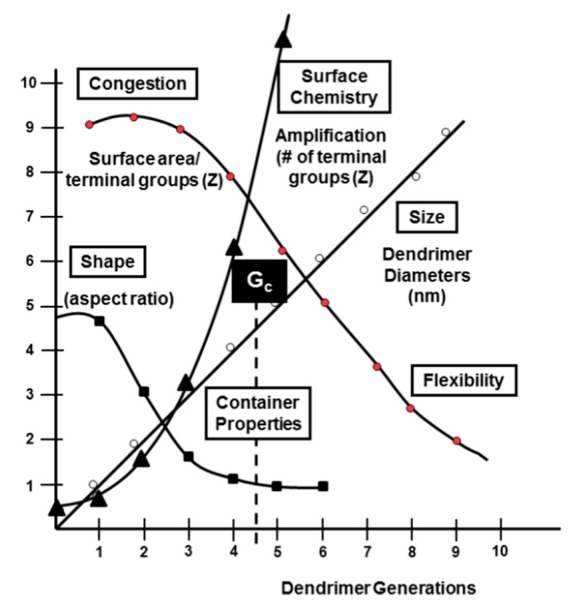
A comparison of emerging nano-periodic property patterns (i.e., endo-and exo-dendritic effects) as a function of: size, surface chemistry amplification and congestion/flexibility. Reproduced by permission from [27]. Copyright 2012 The Royal Society of Chemistry (RSC) on behalf of the Centre National de la Recherche Scientifique (CNRS) and the RSC.

Examination of specific PAMAM dendrimer CNDPs (i.e., generational size, surface chemistry amplification and congestion level) as a function of generation revealed a unique interplay of these generation-dependent nanoperiodic property patterns defining a region; namely: **Gc** bounded by a discrete range of generation levels (i.e., G3–6). It was presumed that the **Gc** region represented generational levels might be expected to exhibit optimum container properties and hence manifest unimolecular micelle behavior (Figure 27).

**Figure 28:**
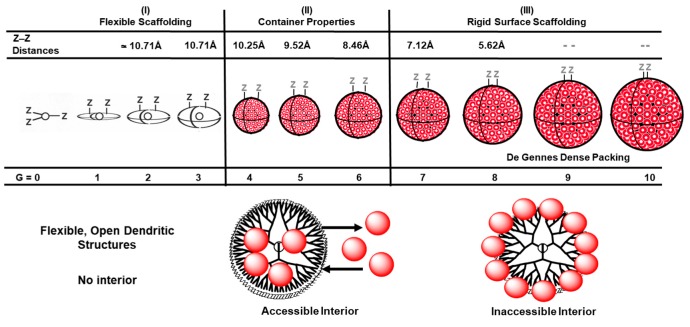
Congestion-induced dendrimer shape changes (I–III) with development of nanocontainer properties for a family of [core: 1,2-diaminoethane]; (4→2); *dendri-*poly(amidoamine)–(NH_2_)_Z_}; (G = 0–10) (PAMAM) dendrimers: N_c_ = 4; N_b_ = 2; where, Z–Z=distance between surface groups as a function of generation. Reproduced by permission from [90]. The Royal Society of Chemistry (RSC).

This assumption was demonstrated to be correct after these predictions were confirmed experimentally and are illustrated in Figure 28. As shown, early generations (i.e., G0–3) may be characterized as open and flexible with complete accessibility due to lower congestion; whereas, G3-6 begin to develop congestion-induced spherical conformations with more discrete accessibility to the interior that possess container properties suitable ideal for the encapsulation of many smaller guest molecule. However, beginning with ~G7, enhanced surface congestion prevents most small organic molecules and metal salts from entering the dendrimer interior. The importance of dendrimer generation levels (i.e., size, surface congestion) [10] leading to effective dendrimer container properties and unimolecular encapsulation behavior has been reviewed extensively elsewhere [89].

**Figure 29:**
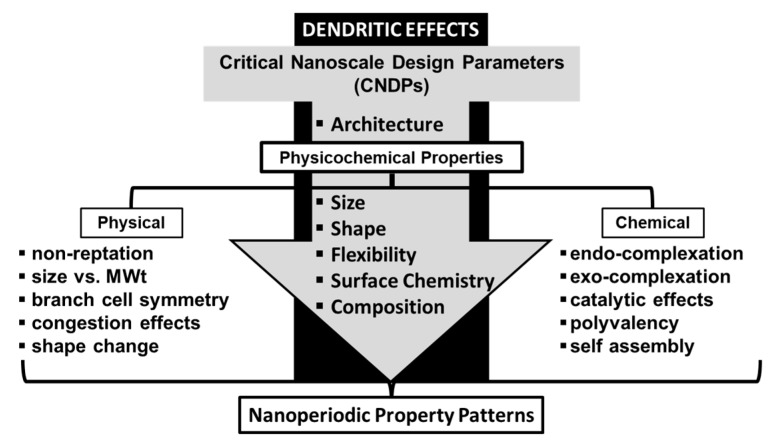
The relationship of dendritic effects to critical nanoscale design parameters (CNDPs) that include: (1) size, (2) shape, (3) surface chemistry, (4) flexibility/rigidity, (5) architecture and (6) elemental composition and the emergence of nanoperiodic property patterns based on these relationships. Reproduced by permission of [27] The Royal Society of Chemistry (RSC) on behalf of the Centre National de la Recherche Scientifique (CNRS) and the RSC.

The early dendrimer literature contains many examples of systematic periodic dendrimer property trends that varied as a function of generational level. These trends were not specific to a certain dendrimer family, but were observed for all dendrimer types. Initially, these periodic trends were referred to as dendritic effects. However, they are now recognized as pervasive nanoperiodic property patterns [27] analogous to traditional elemental periodic property patterns. In fact, these nanoperiodic property patterns are observed for all discrete nanoparticles including dendrimers and have been found to be related to six critical nanoscale design parameters (CNDPs); namely: (1) size, (2) shape, (3) surface chemistry, (4) rigidity/flexibility, (5) architecture and (6) elemental composition [10,27,90,91]. These six dendrimer CNDPs systematically define a wide range of important dendrimer-based nanoperiodic property patterns that heuristically mimic traditional elemental atom property patterns reported by Mendeleev in the 19th Century [92] (Figure 29).

**Figure 30:**
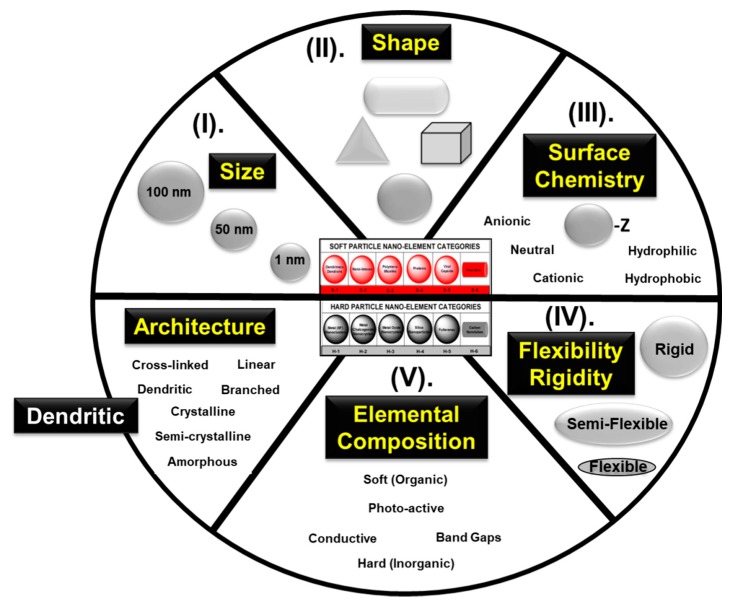
Engineering critical nanoscale design parameters using dendritic architecture, (**I**) size, (**II**) shape, (**III**) surface chemistry, (**IV**) flexibility/rigidity, (**V**) architecture and elemental composition.

Engineering CNDPs for dendrimers (Figure 30) and other discrete, well-defined nanoparticles has become a widely recognized strategy for optimizing desirable properties for both life science [93,94], as well as other nanomaterial applications [95]. Using this approach, selected examples are presented using dendrimer-based CNDP engineering for optimizing drug/guest encapsulation and solubilization in Section 5.

**Figure 31:**
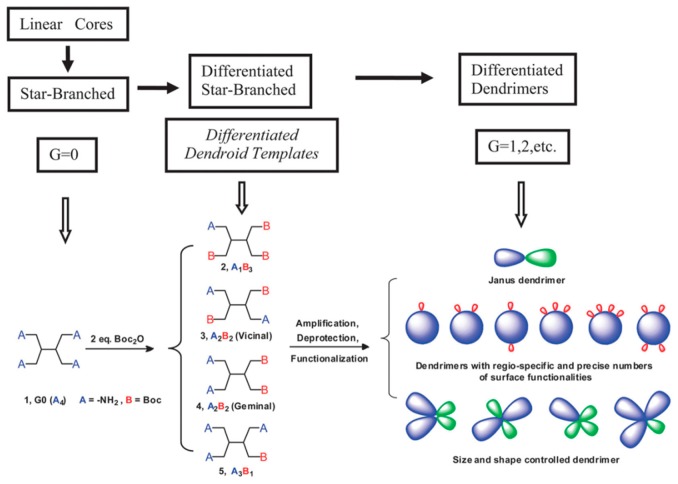
Schematic illustration of the differentiated dendroid template (DDT) strategy for producing higher generation regio-differentiated dendrimers. Reproduced by permission of [96]. Copyright 2013 The Royal Society of Chemistry (RSC) on behalf of the Centre National de la Recherche Scientifique (CNRS) and the RSC.

More specifically, an approach developed by Huang and Tomalia [96] referred to as the differentiated dendroid template (DDT) strategy demonstrates the degree of control that is possible over the synthetic construction of certain dendrimer structures; wherein, essentially all six CNDPs may be systematically engineered and tuned. This approach first involves the synthesis of partial Boc-protected, G0, PAMAM structures using sub-stoichiometric reaction conditions. These partial Boc-differentiated dendroid templates were readily isolated by silica gel chromatography and then used as regio-differentiated core-type starting intermediates. Using traditional divergent, iterative dendrimer amplification protocols, higher generation differentiated dendrimers could be produced possessing specifically engineered CNDPs including: sizes, shapes, surface chemistries, flexibilities/rigidities, architectures and elemental compositions, as described in Figure 31.

### 3.4. Systematic Characterization of Dendrimer Encapsulation Properties

**Figure 32:**
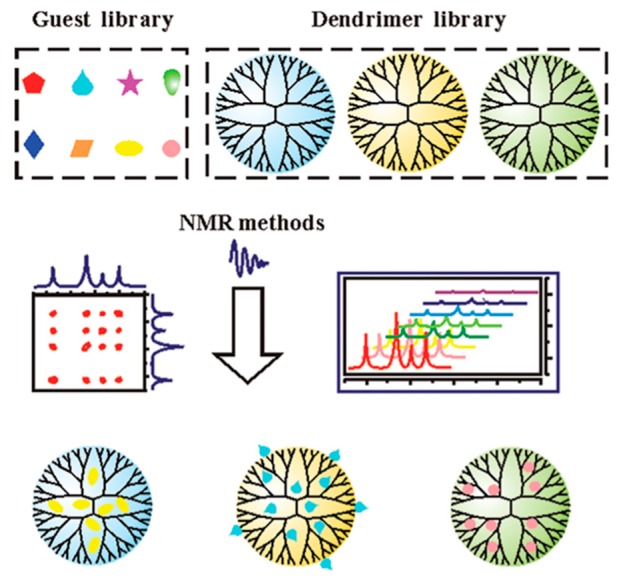
Systematic NMR characterization of dendrimer encapsulation properties as a function of a small molecule guest and dendrimer host library. Reprinted with permission from [97]. Copyright 2012 American Chemical Society.

An excellent review by Cheng et al. [97] provides a comprehensive survey of key NMR protocols used to systematically characterize solubilization/encapsulation interactions occurring between large libraries of guest molecules and dendrimers (Figure 32). This work describes a variety of NMR protocols for analyzing a wide range of guest–host interactions including; chemical shift titrations, saturation transfer difference (STD), spin relaxation, DOSY, NOE, PGSE diffusion measurements to mention a few. For example, in a typical STD-NMR experiment, unique ^1^H-saturation transfer differences from a receptor (i.e., host) to a ligand (i.e., guest molecule) may be used to diagnostically identify specific binding of the guest to the host. Keeping in mind that small molecule guests usually exhibit distinct chemical shifts in high-resolution ^1^H-NMR spectroscopy, STD technique may be used to simultaneously screen the interactions of several guests toward a host from a mixture; as such, STD-NMR is widely used in high-throughput drug screening. The STD signal intensity is related as a function of the binding affinity between the guests and the host, thus allowing quantitative insights into the competitive binding of multiple guest molecules with a target host.

**Figure 33:**
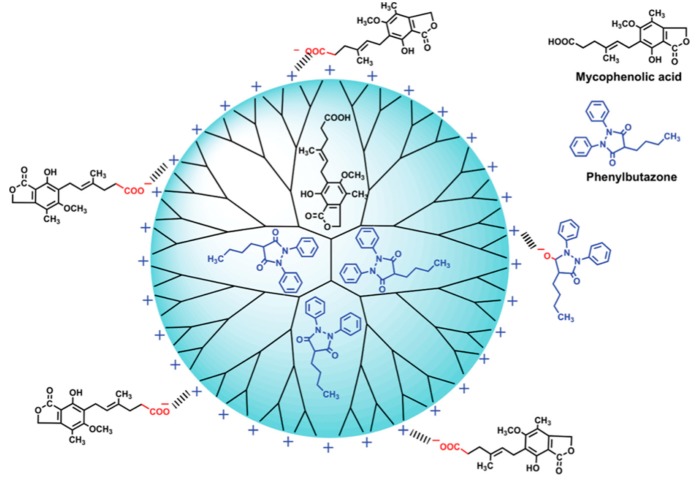
Interaction models for a ternary host–guest system consisting of dendrimer/mycophenolic acid/phenylbutazone. Reprinted with permission from [98]. Copyright 2009 American Chemical Society.

More specifically, these protocols have shown that a variety of factors such as hydrophobicity, size, pKa value, charged groups, and spatial hindrance effect of the guests can determine the localization of guest molecules either on the surface and in the interior pockets of the host. For example, in a ternary host−guest system consisting of dendrimer/mycophenolic acid/ phenylbutazone, much more phenylbutazone molecules localized in the interior pockets than mycophenolic acid, while more mycophenolic acid bound on the surface of dendrimer by ionic interactions than phenylbutazone (Figure 33). This may be attributed to the fact that ionic interaction between the dendrimer surface and mycophenolic acid (−NH_3_+/−COO−) is stronger than that between dendrimer and phenylbutazone (−NH_3_+/−CO−) with higher steric hindrance around the charged groups; wherein, phenylbutazone with two aromatic rings and an aliphatic chain is much more hydrophobic than mycophenolic acid.

**Figure 34:**
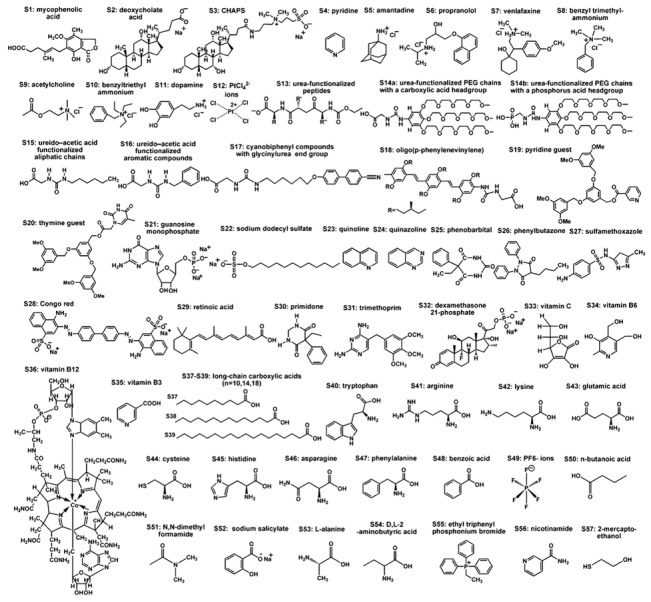
Various guest molecule structures examined using NMR methodologies as described by Cheng et al. Reprinted with permissions from [97]. Copyright 2012 American Chemical Society.

These techniques were applied to a wide range of dendrimer-based host–guest systems that have that have been reported in the past two decades; wherein, the diversity of guest molecules examined are as described in Figure 34.

**Figure 35:**
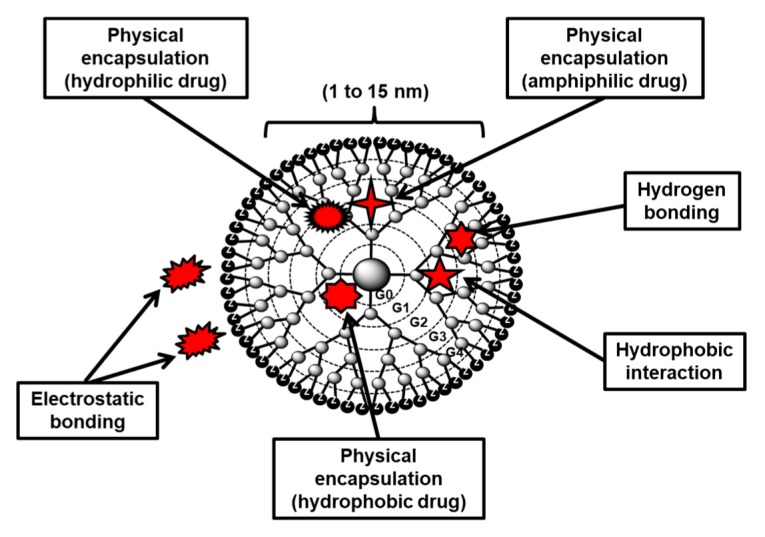
Various generic dendrimer host–guest molecule interactions identified to occur at the exterior or within the interior domains of a dendrimer using recognized NMR protocols.

A wide range of guest–host interaction relationships have been identified using these systemic NMR protocols and many are briefly outlined, as illustrated in Figure 35. Accumulative thermodynamic energies associated with these specific interior interactions ultimately determine whether incarceration is favored over release of the guest to the external bulk phase. Therefore, understanding specific dendrimer host–guest interactions provides invaluable insights for engineering dendrimer CNDPs as a strategy for optimizing important dendrimer host functions for such applications as catalytic activity, sustained/controlled guest release behavior or increased aqueous solubility/stability, as described later in Section 5.

#### 3.4.1. Supramolecular Guest Encapsulation/Aggregation or Covalent Conjugation for Enhanced Aqueous Solubility

The interiors of many available dendrimers are generally found to be more hydrophobic relative to designed hydrophilic surface groups required to attain water solubility. As such, hydrophobic drug interactions in these interior cavities with or without hydrogen bond interactions may define suitable unimolecular micelle features for encapsulating hydrophobic drugs/bio-actives [99]. Generally speaking, higher generation dendrimers exhibit a greater capacity (i.e., interior void space) to encapsulate hydrophobic guests. However, with generational amplification of branch cells and surface groups a limit is reached; wherein, the capacity for encapsulation is significantly reduced due to the de Gennes dense packing and structural-back folding [26,27,63,99,100,101]. Guests/drugs may interact non-covalently with dendrimers by unimolecular micelle encapsulation into interior void spaces or by association with dendrimer surface groups. Driving forces for these interactions usually involve hydrogen bonding, van der Waals interactions or electrostatic charge attraction between dendrimers and API-type guest molecules [102]. Often ionic surface interactions may contribute more to hydrophobic drug solubility enhancement than interior encapsulation and in some cases, encapsulation is dependent on associated surface binding. The interactions involving both ionic surface binding, as well as dendrimer-based interior host–guest systems will depend on the presence of ionic, polar, hydrophobic/hydrophilic and hydrogen-bonding moieties, pKa values and relative sizes of both dendrimers and guests. Interactive guest–host forces within the dendrimer interior versus guest interactions with exterior bulk solution may be dramatically influenced by external parameters such as pH, polarity, and temperature and may be used to modulate encapsulation and release of guests/drugs from these dendritic structures [57,101].

**Figure 36:**
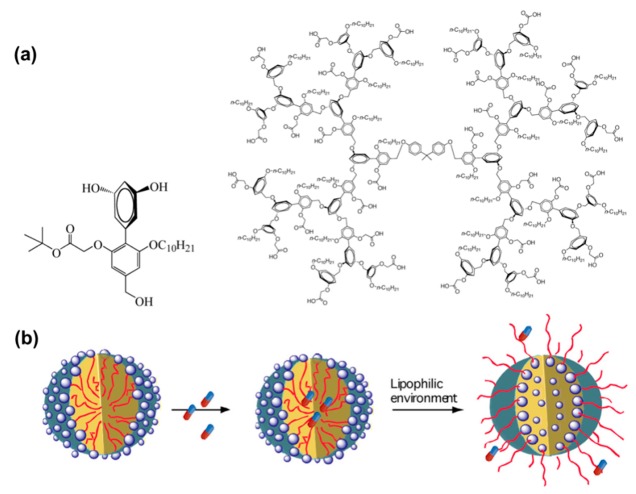
(**a**) The amphiphilic AB_2_ monomer (i.e., on the left) was used to construct the final biaryl amphiphilic dendrimer structure (i.e., on the right). (**b**) Environment-dependent conformational micellar changes in biaryl-based amphiphilic dendrimers leading to potential new targeted drug release systems. Reprinted with permission from [103]. Copyright 2005 American Chemical Society.

Thayumanavan et al. [68] describe the synthesis of an interesting new class of facially amphiphilic dendrimers based on a biaryl AB_2_ monomer design; wherein, hydrophilic (i.e., a carboxylic group) and hydrophobic (i.e., long carbon chain) moieties of the AB_2_ building block are positioned in an orthogonal plane containing the AB_2_ functionalities, as illustrated in Figure 36a. As such, the final dendrimer structure can then switch from a normal to a reverse micelle conformation as a function of solvent or surrounding environmental polarity. This was demonstrated by the amount of either polar or non-polar dyes that could be encapsulated in various polar environments. As shown in Figure 36b, the conformation of these biaryl dendrimers can switch from regular micellar structures to reverse micellar structures by a simple change in polar environment. This polarity stimulated micelle reversal property is expected to facilitate the release of encapsulated hydrophobic drug upon reaching a targeted cells by adapting an inverted micellar strucure as shown. These unique dendrimer-based unimolecular micelle structures as well as many others have been reviewed extensively by Thayumanavan et al. [103] elsewhere.

**Figure 37:**
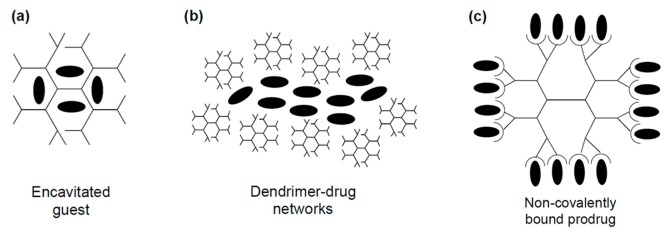
(**a**) Non-covalent unimolecular micelle behavior involving dendrimer-drug interactions, (**b**) non-covalent dendrimer aggregate-drug conjugates involving dendrimer-drug interactions and (**c**) non-covalent dendrimer-drug conjugates involving ionic surface interactions. Reprinted with permission from [111]. Copyright 2002 Elsevier.

As described earlier in Figure 28, the higher generation dendrimers (i.e., G3–6) are more suitable for unimolecular micelle-guest encapsulation complexes (Figure 37a); whereas, lower generation dendrimers (i.e., G0–3) are more inclined to form aggregated dendrimer guest-type complexes (Figure 37b)**.** However, within the context of this aggregated dendrimer-drug concept, there are many reports of dendrimer-drug complex size measurements that suggest dendrimer aggregation species may be [104,105,106,107,108,109,110] involved in drug solubilization versus unimolecular micelle behavior.

**Figure 38:**
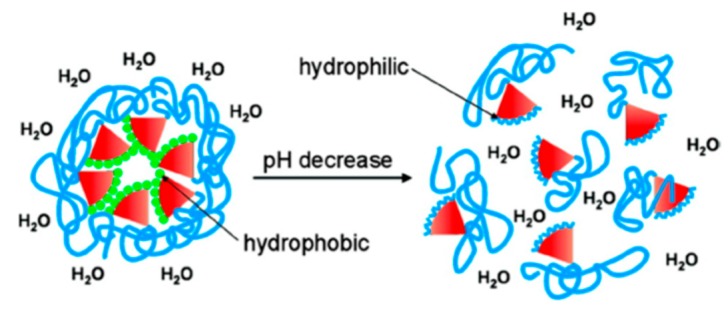
Linear-dendritic architectural copolymer micellar disassembly triggered by pH change. Reprinted with permission from [112]. Copyright 2004 American Chemical Society.

More specifically, Fréchet et al. [112] and others [103] have shown that linear-dendritic architectural copolymers can be very effective at solubilizing drugs by forming dendrimer aggregate-drug complexes that may be triggered to disassemble by a simple change in pH as shown in Figure 38.

**Figure 39:**
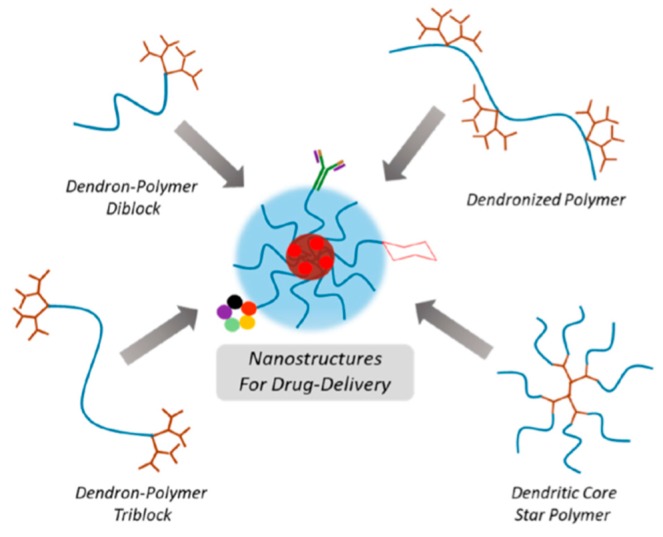
Overview of various linear-dendritic architectural copolymers that undergo supramolecular assembly to form a broad diversity of nanoscale drug delivery vehicles [113].

A wide range of linear-dendritic architectural copolymer structures have been reported to undergo supramolecular assemble to form a diversity of soluble dendritic nanostructures suitable for drug delivery (Figure 39). This very active area has recently been reviewed recently by Bolu and the Sanyal et al. [113].

Although non-covalent drug complexation/entrapment in dendrimers is the preferred technique for solubilizing many drugs, this approach has some limitations. For example, exposure to biological fluids (i.e., ionic electrolytes) may disrupt interior guest–host interactions leading to uncontrolled release of the guest/drug from the dendrimer interior pockets/cavities [59,114,115]. If such a pre-release of encapsulated drug can be avoided or at least minimized, physical drug encapsulation (i.e., unimolecular micelles) remains an attractive approach. That withstanding, there are always concerns by some over potential drug leakage with potential release to off-target sites using unimolecular micelle behavior principles [116].

**Figure 40:**
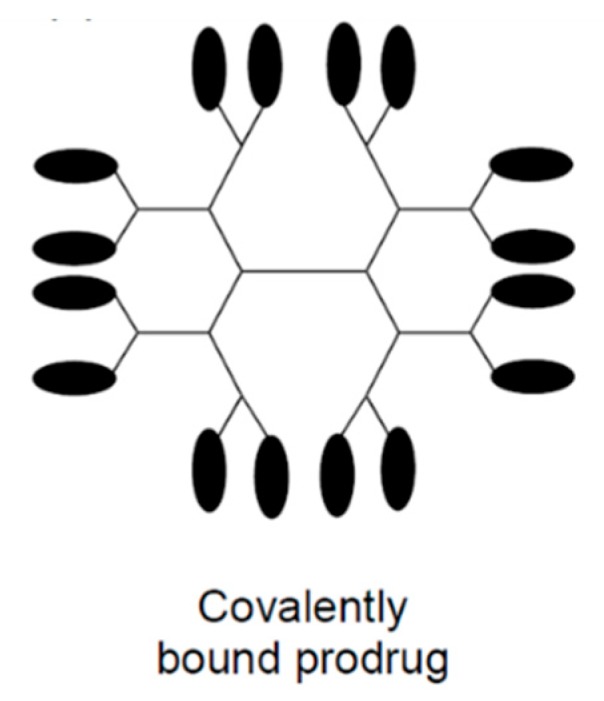
Soluble covalent dendrimer-drug conjugates suitable for targeting and other modifications to optimize circulation residency time and biocompatibility. Reprinted with permission from [111]. Copyright 2002 Elsevier.

As such, many contend that the most acceptable and successful dendrimer-based drug delivery vector will ultimately be a targeted, covalently bound drug-dendrimer conjugate as illustrated in Figure 40.

However, there is broad consensus that successful covalent dendrimer-drug conjugates will have to be designed, engineered and produced according to a strict list of criteria as outlined below:

- Linkers must display very rapid drug release in the disease site.
- Linkers must allow liberation of unmodified yet active drug.
- Must have long in vivo circulation characteristics.
- Highly specific and active disease site targeting features.
- Limited bio-distribution to any off-target organs.
- Suitable nanoscale sizes (i.e., <10–15 nm) for deep tumor penetration and renal excretion.
- Acceptable conjugate excretion mode, preferably via the kidney.
- Good aqueous solubility.

Redox-sensitive dendrimer components based on disulfide linkages have been designed into more advanced dendrimer vectors or their conjugates to provide unique drug-delivery systems exhibiting amplified release properties in combination with encapsulated drugs. These so-called “smart systems” may be activated in vivo as they encounter certain enzyme levels as a function of disease sites, certain organs or physiological conditions in a patient. This “self-immolative strategy” and others have been extensively reviewed elsewhere [117].

The important role of non-covalent (i.e., supramolecular) dendrimer-drug interaction, as well as dendrimer-drug conjugates in a wide range of nano-therapeutic drug delivery systems has received substantial attention during the last decade. This area has been reviewed extensively elsewhere [60,97,118,119,120,121,122].

## 4. Enhanced Solubilization of Active Pharmaceutical Ingredients with Dendrimers

### 4.1. The Significant Role of Dendrimers in the Delivery of APIs

According to *Lipinski’s Rule of Five*, it is well-known that pharmaceutically active ingredients (APIs) tend to be water-insoluble and generally possess moderate lipophilic properties [123]. Therefore, it is not surprising that poor drug solubility is considered one of the major challenges facing all drug delivery systems. Even a decade ago, it was estimated that approximately $65 billion in drug revenues/year were derived from APIs exhibiting suboptimal bioavailability due to marginal water solubility properties. Most notable, is the oral drug delivery market (i.e., $35 billion/year), which is growing at 10% per year. Nearly 40% of all newly developed APIs are rejected without further development by the pharmaceutical industry, largely due to low water solubility properties. An additional 17% of all launched APIs are found during preclinical development to exhibit sub-optimal performance due to poor water solubility features [120].

**Figure 41:**
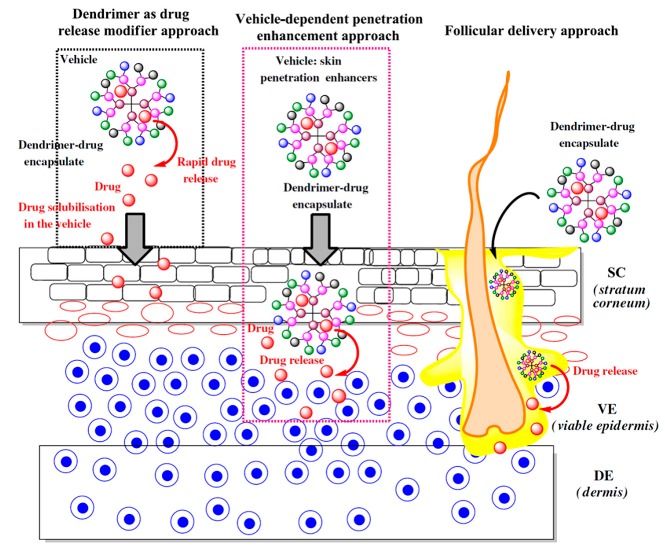
A systematic illustration of possible mechanisms; wherein, dendrimers may act as a release modifier to enhance skin absorption. When applying aqueous solutions of drug-loaded dendrimer to the skin, the solubilized form of the drug enables rapid release with high thermodynamic activity to boost drug permeation. Poor drug solubility in water limits dissolution, resulting in low drug activity and inefficient permeation. Adapted from [124] The Royal Society of Chemistry. Reprinted with permission from [126]. Copyright 2012 Elsevier.

As such, an effective API must manifest a certain level of hydrophilicity or polarity to be water-soluble. In concert, these APIs must exhibit a certain level of lipophilicity/apolarity in order to cross necessary lipophilic cell membranes and provide acceptable bioavailability to targeted disease sites. Therefore, it is not surprising that dendrimer-based drug solubilization/stabilization involving either supramolecular assemblies or covalent conjugates is an area of high interest and activity. Since 65% of the human body is composed of water, it is readily apparent why drug water solubility is a critical property challenging all APIs. In fact, the use of dendrimers for transdermal applications represents a specialized strategy [124] that overcomes this challenge by utilizing the principles of dendrimer-enhanced drug solubility, as illustrated in Figure 41. The importance of engineering dendrimer CNDPs for enhanced transdermal drug delivery applications has been reviewed by Hong et al. [125].

Although a remarkable range of water-insoluble guest molecules have been solubilized by dendrimer-based principles described earlier in Section 3, it is truly amazing how these dendrimer-drug enhancement activities have been applied to so many well-known FDA-approved APIs [127]. In addition to traditional formulation strategies, new parameters are being considered such as dendrimer core types, interior compositions, generation levels, surface chemistries, dendrimer surface PEGylation to produce new dendrimer core:PEG shell architectures to mention a few. A variety of dendrimers have been used in preclinical/clinical studies to enhance solubility, sustained/controlled release, bioavailability, bio-distribution, toxicity management and targeted delivery.

This next section overviews selected examples of API structures that have been encapsulated, complexed or conjugated onto dendrimers according to specific disease or therapy types (Table 2, Table 3, Table 4, Table 5 and Table 6).

#### 4.1.1. Anti-Cancer Drugs

#### 4.1.2. Anti-Inflammatory Drugs

#### 4.1.3. Anti-Hypertensive Drugs

#### 4.1.4. Ocular, Retinal Drugs

The protein, neurotrophin-4 (NT4), has traditionally been regarded as a promising therapeutic agent for the treatment of damaged retinal pigment epithelium cells. Unfortunately, administration of naked NT4 protein without a carrier was found to be ineffective due to rapid degradation in biological media, leading to short protein half-life and low bioavailability. Insights into these degradation/delivery issues were gained by in vivo administration of this protein as a polyvalent charged (i.e., negative or positive) NT4-poly(amidoamine) (PAMAM) (G6) dendrimer complex. These studies revealed that the NT4-PAMAM (G6) dendrimer complexes may be used for continuous NT4 delivery by enhancing protein distribution into mouse vitreous, as well as damaged retina tissue over a 28-day period after injection. More specifically, injection of negatively charged NT4-dendrimer complex enhanced NT-4 delivery to vitreous bodies of injured mouse eyes up to day seven post-injection compared to PAMAM dendrimer alone and then declined. Whereas, positively charged NT4-dendrimer complex increased NT4 levels in impaired retinas compared to controls up to seven days post-injection and then declined. Analyses revealed higher levels of protein transport into the vitreous body compared to the damaged retina layers; wherein, NT-4 levels were found to be 32x higher in the vitreous body than the damaged retina tissue. It is hypothesized that this hampered retina penetration may result from adverse hydrophobic/electrostatic interactions upon encountering negatively charged retinal cell membranes. After 28 days, all protein-dendrimer complex levels were observed to decline to undetectable levels in the eyeballs, vitreous bodies and retina tissue presumably due to associated phagocytosis activity.

#### 4.1.5. Anti-Oxidant Compounds

Resveratrol is a stilbene compound known for its anti-oxidant/anti-ageing properties that suffers from poor water solubility [172] developed a G4, PAMAM dendrimer formulation for transdermal applications with improved water solubility, stability and skin penetration.

#### 4.1.6. Anti-Microbial Drugs

## 5. Engineering Critical Nanoscale Design Parameters: A Proven Strategy for Obtaining Optimum Dendrimer Performance Properties

It is notable that nearly a decade ago a major effort concerning the safe/effective use of nanoparticles in nanomedicine applications [178] had begun to focus on critical nanoparticle CNDP’s implicated in bio-physico-chemical interactions occurring at all nanobiological interfaces. This activity appeared nearly simultaneously with similar nanoparticle CNDP concepts advanced by Tomalia et al. [90,91,92,179,180,181].

**Figure 42:**
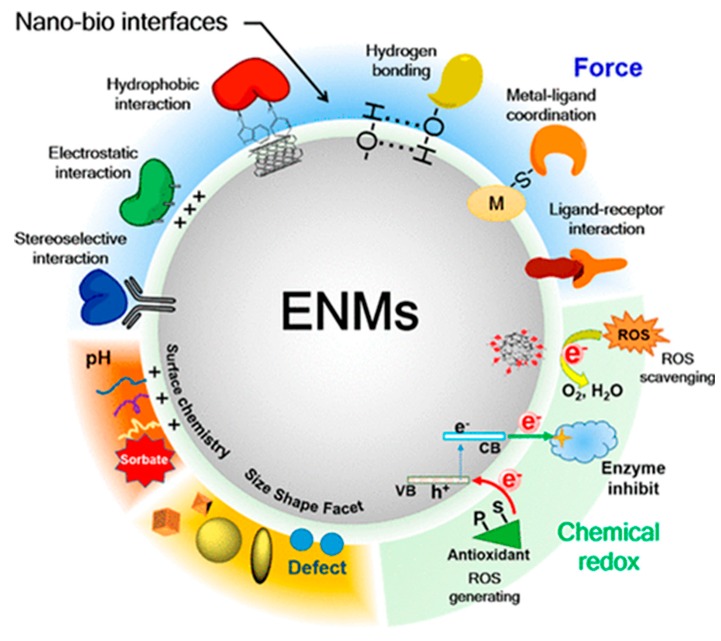
Main driving factors (i.e., CNDPs) at the nano–bio interface of a nanoparticle influencing development of nanomedcines. Reproduced with permission from [182]. Copyright 2019 American Chemical Society.

The importance of understanding and controlling active parameters at these nano-interfaces is apparent when considering fundamental nano-bio-forces associated with NP: protein corona formation, membrane interactions, cellular entry-departure and excretion-filtration events to mention a few. Nel et al. hypothesized that predictable structure–activity relationships should exist as a function of certain nanoparticle features such as: size, shape, surface chemistry, roughness and surface coating. This early hypothesis invoked at least three of the six CNDPs that are now known to define and influence those important nano-interfacial interactions between nanoparticles and biological substrates [178]. More recently, Chen et al. [182] have reviewed the important role that nanoparticle CNDPs play in the nano–bio interactions of nanomedicines as outlined in Figure 42.

It is now widely recognized [180,183,184,185] that all physico-chemical properties of dendrimers and other well-defined nanoparticles are strictly controlled by six nanoscale design parameters (CNDPs); namely: (1) size, (2) shape, (3) surface chemistry, (4) flexibility/rigidity, (5) architecture and (6) elemental composition. These six CNDPs associated with all well-defined hard/soft nanoparticles may be used for the systematic design and engineering of new nanoparticle properties [186], as well as a premise for unifying nanoscience [90,91,92,184]. For example, CNDP engineering of well-defined soft nanoparticles such as dendrimers has been used successfully for optimizing and designing many new nano-therapies [93] and are being used for optimizing promising dendrimer-based cancer drug-delivery systems (i.e., DEP^®^) currently under development by Starpharma Ltd. (Melbourne, Australia).

**Figure 43:**
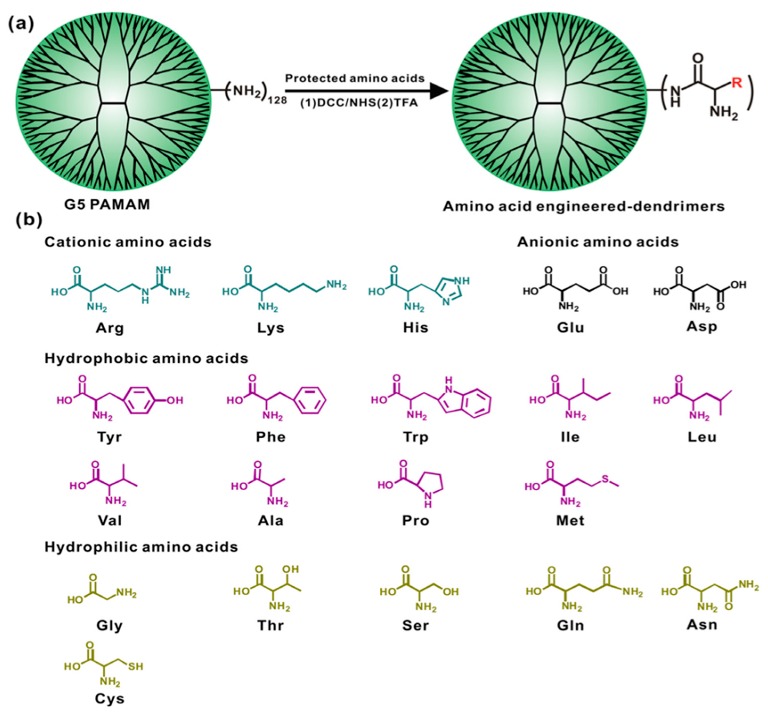
(**a**) Synthesis of G5, PAMAM dendrimers engineered with twenty common amino acids using a facile condensation reaction and (**b**) structures of the conjugated amino acids. The amino acids are divided into cationic, anionic, hydrophobic and hydrophilic amino acids according to the residue group. Reprinted with permission from [94]. Copyright 2016 Elsevier.

The literature contains many diverse examples of dendrimer CNDP engineering. For example, Wang et al. [187] reported dendrimer surface chemistry engineering studies involving the tunability of peripheral dendrimer hydrophobicity. This CNDP modification was demonstrated to allow rapid endosomal escape of drug conjugated dendrimer vectors leading to more effective delivery. More specifically, Wang and Cheng [94] systematically engineered only one CNDP (i.e., surface chemistry) for G5, amine-terminated PAMAM dendrimers with twenty different common amino acids, as shown in Figure 43.

**Figure 44:**
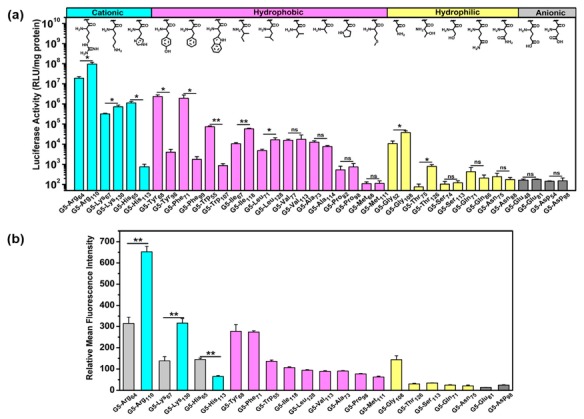
(**a**) The relative luciferase activity of HEK293 cells transfected with various amino acid surface modified G5; PAMAM dendrimers at their optimal N/P ratios for 24 h. Each data point represents mean ± standard deviation (*n* = 3), statistically significant differences analyzed by students’ *t*-test are denoted by *p < 0.05 and **p < 0.01, ns represents non-statistical significance. (**b**) The cellular uptake efficacy of dendrimer polyplexes derived from the various amino acid surface modified G5; PAMAM dendrimers. The polyplexes were labeled with YOOYO-1 and the cellular uptake experiments were conducted on HEK293 cells for 6 hrs. Each data point represents the mean ± standard deviation (*n* = 3), statistically significant differences are denoted by **p < 0.01. Reprinted with permission from [94]. Copyright 2016 Elsevier.

The objective was to assess structure–activity relationships of these surface-engineered dendrimers and develop a nanoscale-quantitative structure–activity relationship (N-QSAR) profile for these dendrimers when used as gene transfection vectors. Typical N-QSAR profiles obtained by this CNDP engineering are as shown in Figure 44. Many of these CNDP principles have recently been used in the development of an in silico model for predicting the intravenous pharmacokinetics of dendrimers based on their structural/ physico-chemical properties [188].

### 5.1. Selected Examples of Engineering Dendrimer CNDPs for Enhanced Guest Solubility/Stability

#### 5.1.1. Engineering Size, Shape, Surface Chemistry, Flexibility/Rigidity, Architecture and Elemental Composition

**Figure 45:**
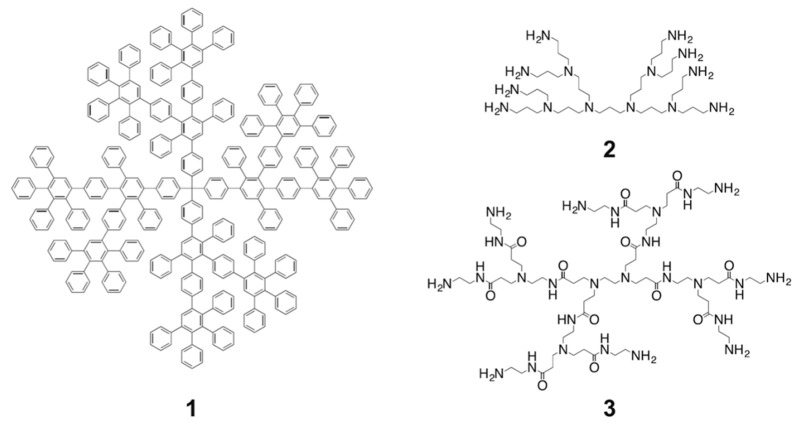
A structural comparison of (**1**) a rigid, shape-persistent (G1); poly(phenylene) dendrimer (PPD) to (**2**) flexible (G1); poly(propylenimine) (PPI) dendrimers and (**3**) flexible (G1); poly(amidoamine) (PAMAM) dendrimer [191].

Seminal synthesis strategies pioneered by the Mullen group (i.e., Max Planck Institute for Polymer Research, Mainz) led to the discovery of a unique class of rigid, aromatic, *shape persistent* dendritic structures referred to as poly(phenylene) dendrimers (PPDs). This dendrimer family was synthesized from a perylene diimide core, using sequential Diels–Alder cycloaddition reactions to yield defect free, monodispersed structures up to 9th generation. Molecular weights as high as 1.9 MDa were confirmed by MALDI-TOF and the shape-persistent PPD structures are compared to flexible PAMAM and PPI dendrimer structures, as illustrated in Figure 45. This work has been reviewed extensively and described elsewhere [189,190].

**Figure 46:**
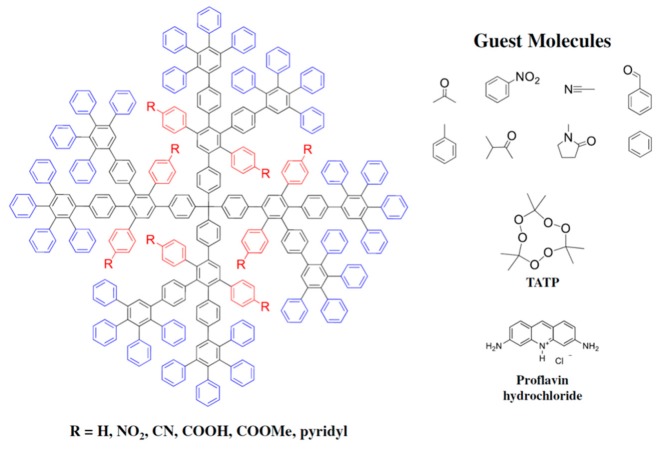
Various poly(phenylene) (PPD) scaffoldings modified with polar groups and non-polar groups to tailor their encapsulation properties toward various polar/non-polar guest molecules. Reprinted with permission from [190]. Copyright 2016 American Chemical Society.

More recently, it has been reported that CNDP engineering of these shape-persistent PPD structures as a function of size, surface chemistry, architecture, etc. [191] allows these dendrimers to function as encapsulation hosts for a broad range of polar and non-polar guest molecules, as described in Figure 46. More specifically, it was found that when R=H, the PPD hosts were able to encapsulate small non-polar molecules (i.e., benzene, toluene, hexane, etc.). On the other hand, it was found that when the PPD scaffold was chemically modified with polar groups (i.e., carboxylic acids, nitriles, nitro or ester groups), they displayed enthalpically driven affinity to encapsulate many of the polar guest molecules involving H-bridges, dipole interactions, and π–π interactions, as shown in Figure 46. For example, a third-generation PPD modified with 12-carboxylic acids was found to encapsulate 3–4 proflavin hydrochloride guest molecules per PPD and function as a nanocarrier by transferring this salt through a non-polar environment while maintaining the chemical structure [192].

#### 5.1.2. Engineering Architecture, Interior Composition, Surface Chemistry

**Figure 47:**
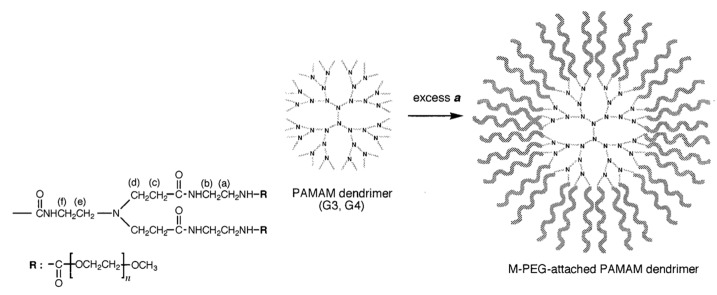
PEGylation of G3, G4; amine-terminated poly(amidoamine) (PAMAM) dendrimers produced dendritic (core)-linear (shell) architectural copolymers with enhanced unimolecular micelle encapsulation properies for guest molecules (i.e., anti-cancer drugs) such as adrimyicin and methotrexate. Reprinted with permission from [114]. Copyright 2000 American Chemical Society.

Kojima et al. [114] enhanced the unimolecular micelle behavior of G3, G4, PAMAM dendrimers by grafting PEG moieties (i.e., Mwt.= 500 and 2000, respectively). This best CNDP modification involved the surface chemistry grafting of PEG; MWt. 2000 to the surface of a G4; amine-terminated PAMAM dendrimer to produce the new dendritic-linear architectural copolymer (Figure 47), which encapsulated 6.5 moles of adrimyicin or 26 moles of methotrexate, respectively. The methotrexate-loaded micelle retained the drug in aqueous solutions with low ionic strength; however, rapid release was noted by increasing ionic concentration.

As described earlier in Section 3.4; Figure 18, grafting hydrophilic PEG moieties onto a hydrophobic dendritic hypercores [58] produced a similar dendritic-linear architectural copolymer that encapsulated pyrene and indomethacin.

#### 5.1.3. Engineering Size, Interior Composition and Surface Chemistry

**Figure 48:**
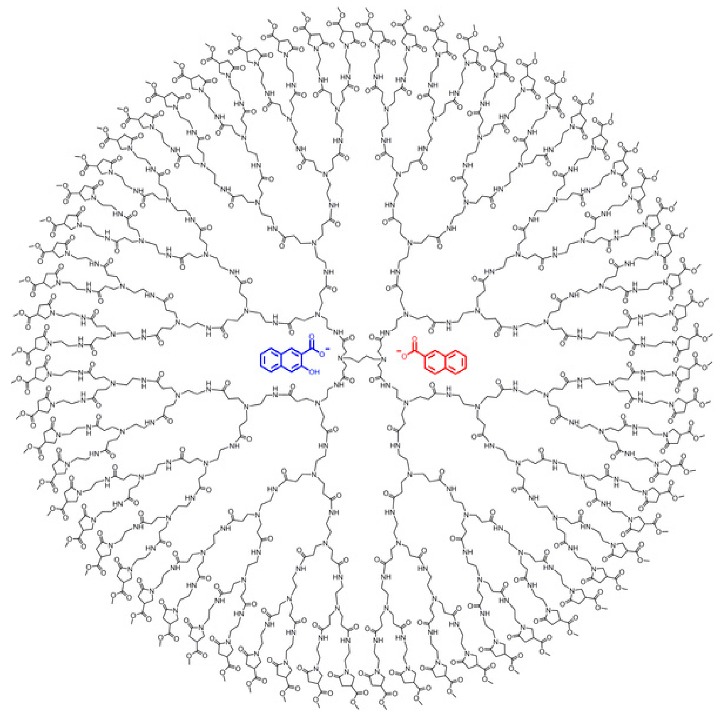
Two anionic naphthanoate guest molecules used as non-steroidal inti-inflammatory models shown to be unimolecularly encapsulated in a 4-(carbomethoxy)-pyrrolidone) functionalized (G4) PAMAM dendrimer host. Reprinted from [193].

The recognition and binding of anions in water is very difficult due to the propensity of water molecules to form strong hydrogen bonds and solvate the anions. As such, the encapsulations of two anionic guest molecules, namely: sodium 2-naphthoate and sodium 3-hydroxy-2-naphthoate, were examined using a 1-(4-carbomethoxy) pyrrolidone, surface-modified PAMAM dendrimer (G4) as the host (Figure 48) [193]. These two guest molecules were used as models/mimics of non-steroidal anti-inflammatory drugs.

Using NMR protocols described by Cheng et al. [97], it was determined that the binding stoichiometry for 3-hydroxy-2-naphthoate involved two strongly bound guest molecules per dendrimer and an additional 40 guest molecules exhibiting weak binding affinity. By comparison, sodium 2-naphthoate showed a weaker binding strength and had a stoichiometry of two guests per dendrimer with no additional weakly bound guests. It was hypothesized that the hydroxyl moiety in the 3-hydroxy-2-naphthoate guest molecule provided additional stabilization of the anionic charge leading to these results.

#### 5.1.4. Engineering Size, Interior Composition, Surface Chemistry

**Figure 49:**
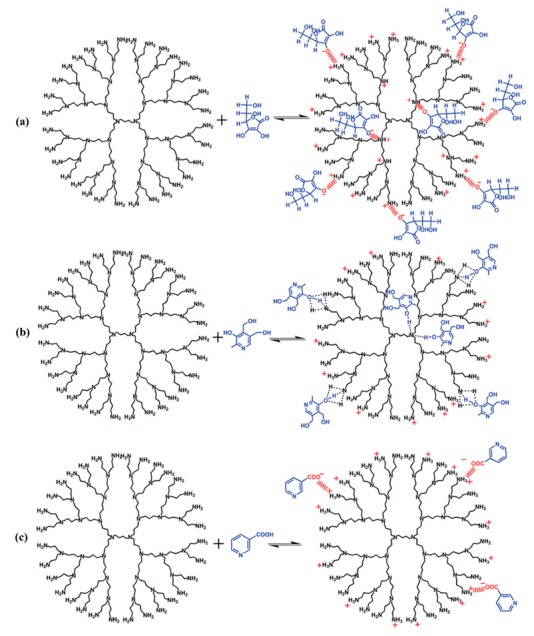
Complexation of G3, Poly(propyleneimine) (PPI) dendrimer with (**a**) Vitamin C, (**b**) Vitamin B6 and (**c**) Vitamin B3. Reprinted with permission from [194]. Copyright 2010 WILEY-VCH Verlag GmbH & Co. KGaA, Weinheim.

Understanding dendrimer-based host–guest interactions at the molecular level can reveal possible mechanisms for the preparation of dendrimer-based interior encapsulation or external type complexes. Astruc et al. [194], reported studies on a series of vitamins; wherein, it was possible to assess these interactions to determine the mode of interaction as a function of the guest molecule structure. Using a specific dendrimer host; namely, G3 poly(propyleneimine) (PPI)-(NH_2_)_32_ dendrimer, core (DAB), size (G3) and surface chemistry (-NH_2_)_32_, it was found that interior encapsulation versus surface complexation varied as a function of the vitamin guest chemical structure (i.e., Vitamin C, B6, B3), as shown in Figure 49. CNDP engineering according to localization details of phenylbutazone (i.e., drug) in the interior cavities and on the surface of a cationic PAMAM dendrimer.

#### 5.1.5. Engineering Architecture-Dendrimer Nanohybrids

**Figure 50:**
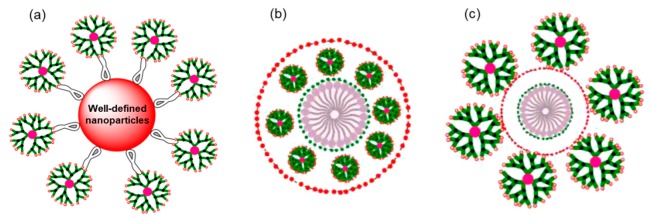
(**a**) Dendrimer-nanohybrids formed by grafting various dendrimers to discrete, well-defined nanoparticles (i.e., gold nanoclusters, quantum dots, carbon nanotubes, etc.), (**b**) Dendrimer encapsulated liposome and (**c**) dendrimer adsorbed onto a liposome. Adapted with permission from [195] with permission of Elsevier.

A new emerging area has focused on the engineering of dendrimer-associated architecture by hybridizing dendrimers with a variety of well-defined, discrete nanoparticles such as liposomes, quantum dots, carbon nanotubes, proteins, metal nano-clusters and microspheres to mention a few (Figure 50a). This new dendrimer nanohybrid architectures offer multiple functionality suitable for theranostic-type applications. A general example of such prototypes is illustrated in Figure 50; wherein, specific examples involving the hybridization of dendrimers with liposomes are shown. This area was recently reviewed by Kesharwani et al. [195].

**Figure 51:**
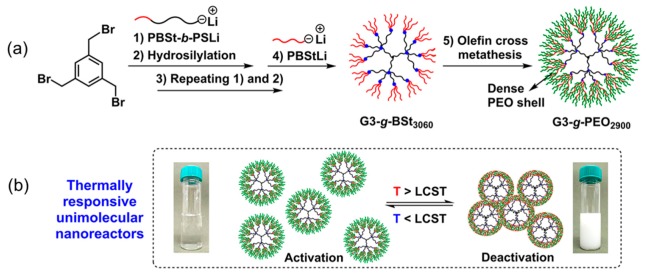
(**a**) Five-step process for synthesis of poly(styrene) interior, PEO exterior unimolecular micelle reactor. (**b**) Thermally responsive unimolecular reactors exhibiting low critical solution temperature (LCST) properties due to PEO periphery. Reprinted with permission from [196]. Copyright 2019 American Chemical Society.

Amphiphilic, dendrimer-like structures were synthesized according to a five-step process described in Figure 51. These amphiphilic structures possess interior hydrophobic poly(stryrene) segments and peripheral hydrophilic poly(ethylene oxide) (PEO) components, which were obtained by iterative anionic polymerization, hydrosilyation coupling followed by olefin cross-metathesis with a PEO macromonomer. These low critical solution temperature (LSCT) thermo-responsive materials behaved as unimolecular, micellar-like reactors, wherein, accelerated catalytic nucleophilic displacements of benzyl halide were observed with at least seven recycles.

## 6. Predictions for the Future

Optimism relative to the future of nanoparticle-based therapies and nanomedicines remain very high with a predicted global market of $350.8 billion market by 2025, according to a recent report by Grand View Research [197].

Similarly, funding support from U.S. government sources continues to remain high with >$130 million spent on nanomedicine alone by the National Institutes of Health (NIH) in 2018. Much of this optimism is based on a widely recognized list of advantages documented for nanoparticle-based therapies/drug-delivery systems compared to traditional drug-delivery protocols [126,198,199,200,201].

Recent issued patents focused on the use of dendrimers for nanomedicine applications through 2014 are extensive and have been reviewed elsewhere [202]. Although the first FDA-approved nanomedicine-based drug, Doxil, was announced in 1995; a PubMed search for published manuscripts associated with the terms nanoparticle-based therapies, nanoparticles for cancer therapy, etc., at that time retrieved only 25 nanomedicine-associated articles. However, recent use of these same search terms (December, 2018) produced >25,000 manuscripts; whereas, only 15 nanoparticle-based medicines have been approved to date. Currently, 75 nanoparticle-based drugs are under clinical investigation, although they face a high probability of failure with a present Phase III success rate of ~14%, largely due to lack of efficacy [203].

These past two decades of dendrimer-based nanomedicine/drug delivery research may be generally characterized as highly empirical. It is generally agreed that more quantitative and systematic strategies are needed for optimizing in vivo efficacy parameters to increase probability for success in the clinic. In Section 2.3, we briefly review preclinical optimization strategies using dendrimer-based CNDP engineering, which is described more extensively elsewhere [204]. Similarly, Kaminskas et al. [188] recently described an in silico model for predicting intravenous pharmacokinetics for dendrimers based on their physicochemical properties. It is claimed that this machine-learning based model is able to accurately predict parameters such as dendrimer half-life clearance, volume of distribution, and dose recovered in the liver and urine. Access to this system is available at a user-friendly web-service and database (located at http://biosig.unimelb.edu.au/dendpoint). This computer-based platform is expected to be used to guide dendrimer design, engineering and refinement prior to time consuming and expensive in vivo testing.

## 7. Conclusions

This account reviews early unreported history and evidence for the significant role that branch cell symmetry plays in differentiating the interior packing modes of dendrimers. These interior packing modes define key physical properties for all dendrimers such as densities, refractive indices and interior porosity/hollowness and ultimately, unimolecular micelle encapsulation (UME) behavior. As such, all known dendrimer structures may be differentiated by branch cell symmetry properties into two primary classifications. The first is Category I: Symmetrical Branch Cell Dendrimers (i.e., dominated by 99.5% of known dendrimers based on citations), largely represented by Tomalia, Vögtle, Newkome, Majoral/Caminade, Fréchet-type structures and are characterized as “non-draining spheres” possessing porous/interior void spaces. The second classification is Category II: Asymmetrical Branch Cell Dendrimers (i.e., currently defined by only 0.5% of all known dendrimer structures based on citation), represented by Denkewalter-type structures. They are characterized as draining spheres possessing solid, tightly packed interiors. These new symmetry classifications provide a background for explaining first examples of Category I based UME behavior, important NMR protocols for systematic UME characterization, application of UME principles to solubilization/stabilization of active/FDA-approved drugs and the engineering of dendrimer CNDPs to produce optimized aqueous drug solubility/stability/delivery properties based on host–guest relationships and UME principles. This account concludes with predicted global nanomedicine activities/markets and high optimism for the anticipated role of dendrimer-based solubilization properties/principles in emerging new life science, drug delivery and nanomedicine applications.

## Figures and Tables

**Table 1:** Historical comparison of Denkewalter, Tomalia and Newkome first divergent, iterative syntheses of dendritic structure.

	Tomalia Type	Newkome Type	Denkewalter Type
Dendritic Topologies	Symmetrical, equal branch cell segments	Symmetrical, equal branch cell segments	Asymmetrical, unequal branch cell segments
1st Patents	U.S. #4,507,466 (1985)		U.S. #4,289,872 (1981)
1st Public Oral Reports	Pauling-Flory Polym. Gordon Conf. (1983) [21]		
1st Publications	1st SPSJ Int. Conf., Kyoto, Japan, Abstract (1984) *Polym. J*. (Tokyo), (1985) (Full Paper) [11]; *Macromolecules* (1986) [22]	*J. Org. Chem*., (1985) (Communication) [24]	
Compositional Types	poly(amidoamines, amides imines, phosphorhydrazones, aryloxys, alkylethers, ureas, esters, siloxanes, arylenes, etheramides, triazines, silanes, carbosilanes, triazoles, thioethers, iminoethers, aryloxyethers, thioether esters, silyloxhydroxyl ethers, alkynes, urethanes, etc.)	poly(amidoethers)	poly(L-lysines)
Total Citations (10/23/19) (Publications & Patents)	45,485 citations (Google Scholar)	35,519 citations (Google Scholar)	282 citations [31]

**Table 2:** Anti-cancer Drugs.

API Structure Loaded	Dendrimer Type (Formulation)	Effects/Results	References
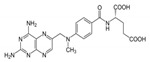 Methotrexate	Triazine (G3)	Encapsulates 3 moles MTX per G3 dendrimer	[128,129]
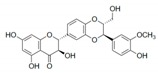 Silybin	PEG-PAMAM (G4)	Encapsulation, high PEG increased solubility	[130]
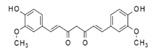 Diferuloylmethane (curcumin)	FA-PAMAM	Encapsulation, increased solubility, targeted delivery	[131]
	HA-PAMAM	Encapsulation, increased cell uptake, reduced toxicity	[132]
	PAMAM-(3.5), (G4)	4.3–5.0 mol/dendrimer	[133]
	PAMAM-meso nanosilica	Encapsulation	[134]
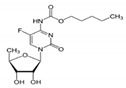 Capecitabine	PAMAM-conjugated	Targeted delivery and lower toxicity	[135]
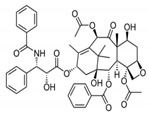 Paclitaxel	Poly(glycerol)	Encapsulation	[136]
	Triazine dendrimer-paclitaxel conjugates	Encapsulation	[137]
	PPI-(CO_2_H)	Encapsulation	[138]
	Antibody conjugate PAMAM-(NH_2_), (G4) lipid hybrid	×465-fold increased solubility and activity	[139]
	PAMAM poly(glycerol)(G4,5)	×400-fold increased solubility	[140]
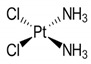 Cisplatin	PAMAM-ester terminated	Solubility enhancement	[24,141]
	PPI-amino butyrate terminated		[142]
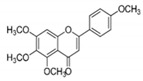 Tetramethylscutellarein	PAMAM-NH_2_, (G4)	Encapsulation Increased solubility	[143]
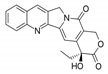 Camptothecin	PAMAM (NH_2_), (G5) PEGylated	Encapsulation Increased solubility	[144]
	Glycerol-succinic acid- poly(ester) dendrimer	×16-fold cell uptake	[73]
	PAMAM dendrimers	Encapsulation	[145]
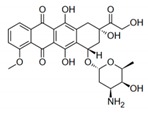 Doxorubicin	PAMAM-(NH)_2_	Enhanced bioavailability	[146]
	PPI-NH_2_, dextrin conjugate	Enhanced solubility	[147]
	PPI-NH_2,_ folate conjugated	Enhanced solubility	[148]
	PPI-NH_2_	6.5 drug/dendrimer	[114]
	PAMAM-(G4)PEG_2000_	91% encapsulated	[149]
	poly(ester); bowtie dendrimer architecture	Conjugated DOX yields Enhanced solubility	[150]
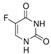 5-Flurouracil	PAMAM-NH_2_, G4	Encapsulation, high loading and	[151]
	PAMAM-CO_2_H, NH_2_	lower toxicity	[152]
	PAMAM-(G4)-PEG_5000_	×12-fold increased solubility	[153]
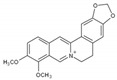 Berberine	PAMAM conjugation & encapsulation	Encapsulation, improved pharmacokinetics	[154]
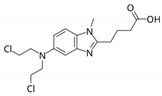 Bendamustine	PAMAM-conjugation, G4	Improved stability/pharmacokinetics	[155]
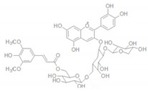 Black carrot anthocyanin	PAMAM-mesoporous silica	Controlled release, lower toxicity, enhanced activity	[156]

**Table 3:** Anti-inflammatory Drugs.

API Structure Loaded	Dendrimer Type (Formulation)	Effects/Results	References
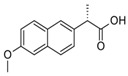 Naproxen	PAMAM-lauryl modification (G0–3)	Enhanced solubility	[157]
	PAMAM-NH_2_ (G4)	×1570-fold increased solubility	[158]
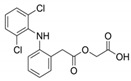 Aceclofenac	PAMAM dendrimers	Solubility enhancement	[159]
	PAMAM dendrimers	Solubility enhancement	[121]
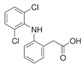 Diclotenac	Citric acid-PEG dendrons (G1–3)	15–33 mol% drug loading	[160]
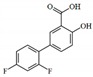 Diflunisal	PAMAM-NH_2_ (G2–4) PAMAM-NH_2_ (G4)	×2.48-fold increase bioavailability ×26-fold increased solubility	[161] [158]
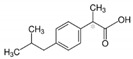 Ibuprofen	PAMAM-NH_2_ (G3,4)	78-IBU mol./dendrimer	[162]
	PAMAM-NH_2_ (G4)	×74-fold increased solubility	[158]
	PAMAM-NH_2_ (G4)	Solubility enhancement	[163]
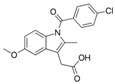 Indomethacin	PAMAM-NH_2,_ -OH, -CO_2_H (G4)	×10–20-fold increased solubility	[164]
	PEHAM, OH, piperazine, PEG_55_ (G1)	×48–136-fold increased solubility	[165,166]
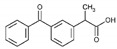 Ketoprofin	PAMAM-NH_2_ (G4)	×19-fold increased solubility	[158]
	PAMAM-NH_2_ megamer hydrogel	Encapsulation and controlled release	[167]
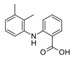 Mefenamic acid	Citric acid-PEG dendrons (G1–3)	27–36 mol% drug loading	[160]
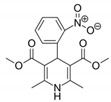 Nifidepine	PAMAM-NH_2_ (G0–3)	G2.5 > G3 > G1.5 (increased solubility)	[168]
	PAMAM ester (G0.5–2.5)	G2 > G0.5 > G1 > G0	[169]
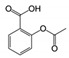 Aspirin	PAMAM methyl ester (G3.5)	30 wt% uptake by encapsulation	[26]

**Table 4:** Anti-hypertensive Drugs.

API Structure Loaded	Dendrimer Type (Formulation)	Effects/Results	References
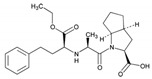 Ramipril	PAMAM hybrid	Encapsulation	[170]
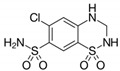 Hydrochlorothiazide	PAMAM hybrid	Encapsulation	[170]
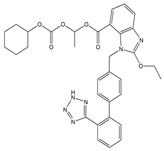 Candesartan cilexetil	PAMAM-NH_2_, -CO_2_H,tris-OH conjugates	Anionic dendrimer enhanced solubility	[171]

**Table 5:** Anti-oxidant Compounds.

API Structure Loaded	Dendrimer Type (Formulation)	Effects/Results	References
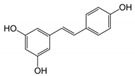 Resveratrol	PAMAM hybrid	Improved solubility	[172]
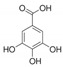 Gallic Acid	PAMAM conjugation	Improved bioavailability	[173]

**Table 6:** Anti-microbial Drugs.

API Structure Loaded	Dendrimer Type (Formulation)	Effects/Results	References
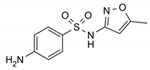 Sulfamethoxazole	PAMAM-NH_2_ (G3)	×40-fold enhancement	[174]
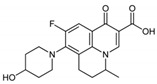 Nadifloxacin	PAMAM (G3–5)	Increased solubility	[175]
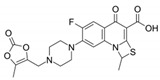 Prulifloxacin	PAMAM (G3–5)	Increased solubility	[175]
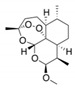 Artemether	PEG-poly(lysine) dendritic micelle (G1.5, G2.5)	×3-15 fold increased solubility	[176]
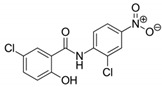 Niclosamide	PAMAM-NH_2_, (G0–3)	×372–6176 fold enhancement	[177]
